# Supplementary material for: Chemically-induced degradation of the endoplasmic-reticulum stress sensor IRE1 by a VHL-recruiting chimera
Source: Nat Commun. 2025 Dec 11;16:11445. doi: 10.1038/s41467-025-66382-8 (PMC12749748; doi:10.1038/s41467-025-66382-8)
Supplement: Supplementary file 1 — Supplementary Information [file 41467_2025_66382_MOESM1_ESM.pdf]

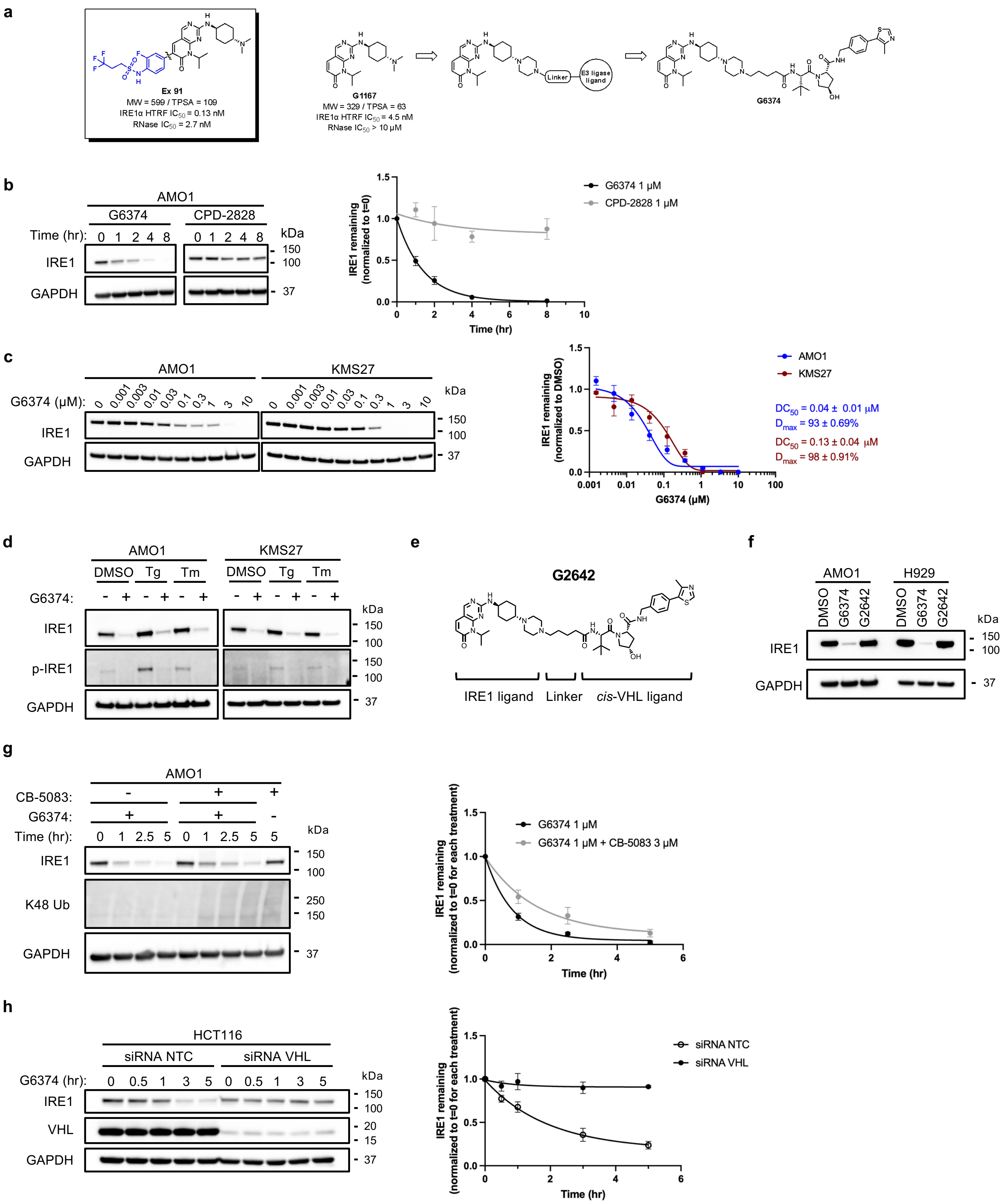

Supplementary Figure 1

i

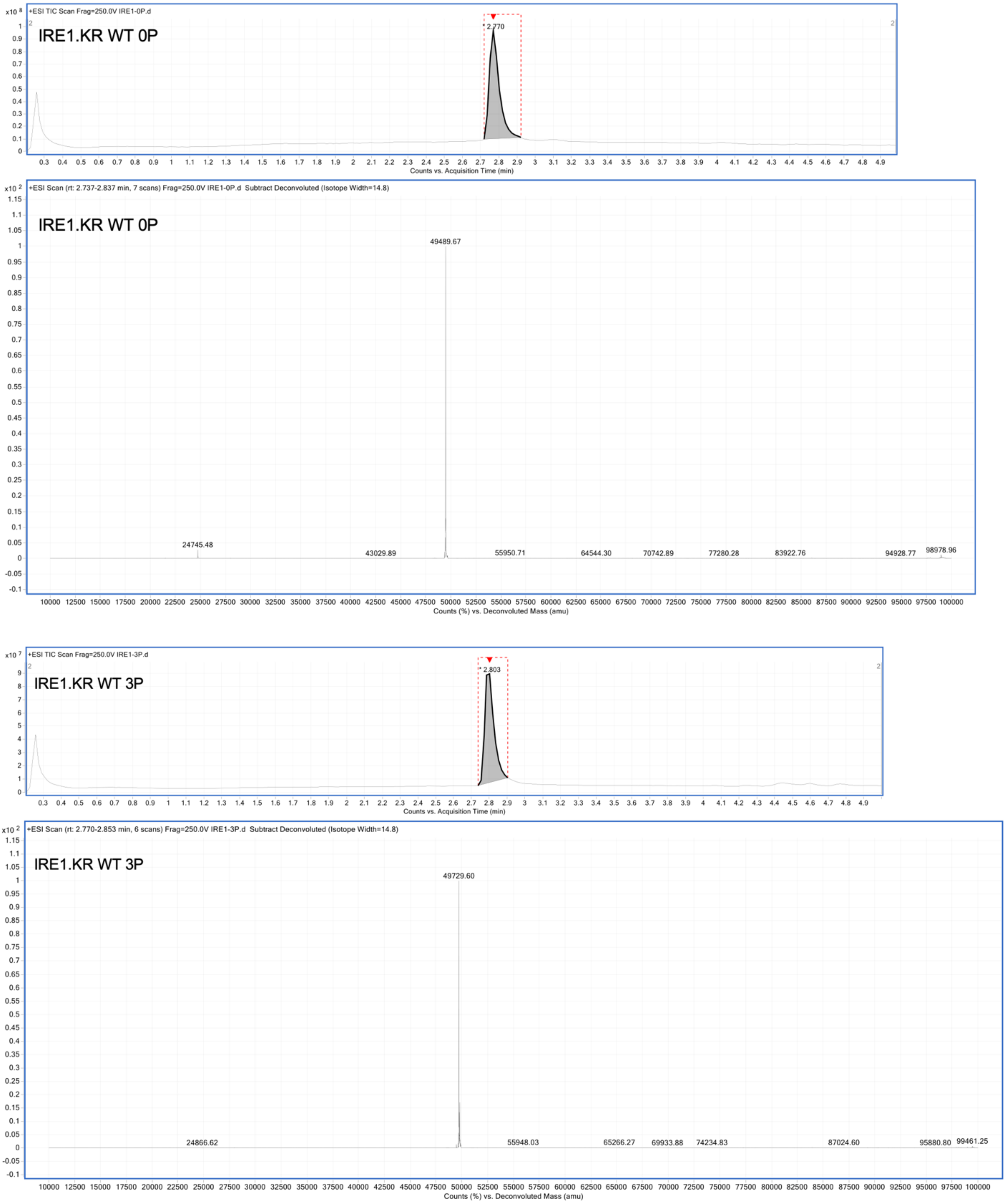

Supplementary Figure 1 (continued)

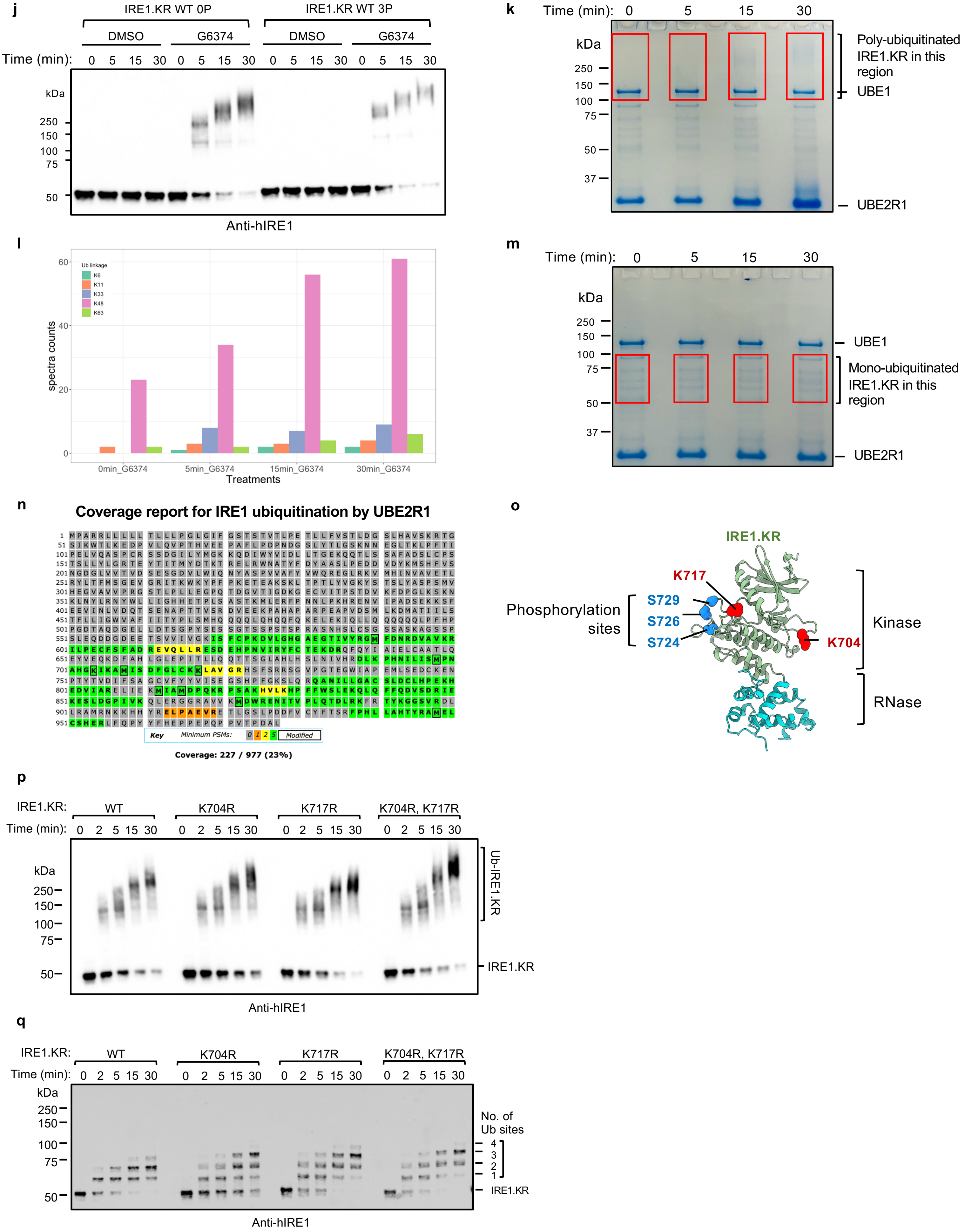

Supplementary Figure 1 (continued)

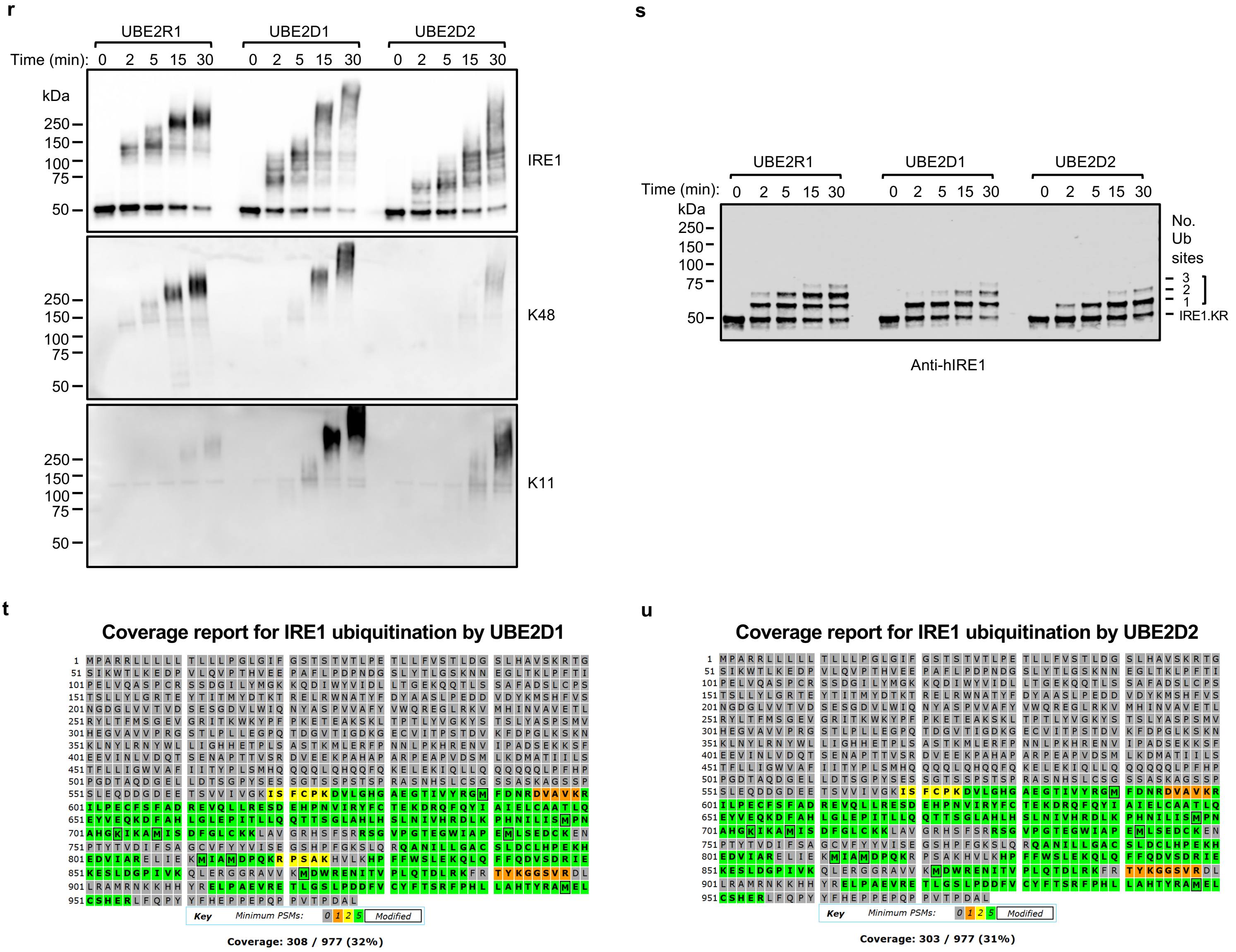

Supplementary Figure 1. G6374 induces efficient ubiquitination and degradation of IRE1. **a.** Development schematic of G6374. **b**. Endogenous IRE1 depletion induced by G6374 or CPD-2828 (1 µM) over a time course in AMO1 cells. **c**. G6374-induced protein depletion of endogenous IRE1 in AMO1 and KMS27 cells. IB analysis of IRE1 over a titration of G6374 (left) with quantification and normalization of the remaining IRE1 protein graphed (right; n=4 biological replicates, mean ± SEM). **d**. G6374-induced protein depletion of endogenous IRE1 (3 µM, 2 hr) in the presence or absence of ER-stress inducers, Tg (100 nM, 6 hr) or Tm (5 µg/ml, 6 hr). **e**. Chemical structure of the epimer control compound for G6374, G2642, which contains a *cis*-VHL ligand. **f**. Endogenous IRE1 protein levels in AMO1 or H929 cells treated with G6374 or G2642 (1 µM, 4 hr). **g**. G6374-induced protein depletion of endogenous IRE1 (1 µM) in AMO1 cells in the presence or absence of the VCP/p97 inhibitor, CB-5083 (3 µM), over a time course. Samples were analyzed by IB against IRE1 and K48-Ub (left), with quantification of the IRE1 protein band graphed (right; n=4 biological replicates, mean ± SEM). **h**. Endogenous IRE1 protein degradation in HCT116 cells (more efficiently transfectable than AMO1 cells) with either siRNA non-targeting control (NTC) or siRNA for VHL (left), with quantification and normalization of the IRE1 protein band graphed (right; n=3 biological replicates, mean ± SEM). **i.** Representative LC-MS chromatograms for IRE1 phosphorylation status. Proteins were injected onto a reverse-phase HPLC analytical column and eluted using a gradient from 95% water-5% acetonitrile to 15% water-85% acetonitrile over 5 min. IRE1.KR 0P – Expected M.W. = 49489 Da. Observed: 49489.67 Da. IRE1.KR 3P – Expected M.W. = 49729 Da. Observed: 49729.60 Da. **j**. *In vitro* ubiquitination analysis of recombinant IRE1.KR having no phosphorylation (0P) or full phosphorylation (3P) on the kinase activation loop with G6374 over a time course. Samples were analyzed by IB against IRE1. **k**. Coomassie Blue staining of proteins from the *in vitro* ubiquitination assay using Ub. The boxed regions were excised for LC-MS/MS analysis. **l**. Distribution of polyubiquitin linkage type on ubiquitinated recombinant IRE1.KR at each time point as determined by mass-spectrometry from **k**. **m**. Coomassie staining of proteins from the *in vitro* ubiquitination assay using Me-Ub. The boxed regions are excised for LC-MS/MS analysis. **n**. Identification of ubiquitination sites on recombinant IRE1.KR by mass-spectrometry from **m**. **o**. Model of IRE1.KR (PDB: 6W3B) labeled with the ubiquitinated lysine residues (red) and known phosphorylation sites in the activation loop (blue). The IRE1 kinase domain is colored in green and RNase domain in cyan. **p**. *In vitro* ubiquitination analysis for recombinant IRE1.KR WT or ubiquitination site mutants over a time course using Ub. Samples were analyzed by IB for IRE1. **q**. *In vitro* ubiquitination analysis for recombinant IRE1.KR as in **p** but using Me-Ub instead of Ub. Samples were analyzed by IRE1 IB. **r**. *In vitro* ubiquitination analysis for recombinant IRE1.KR with different E2 ubiquitin conjugating enzymes over a time course using Ub. Samples were analyzed by IB for IRE1, K48-polyubiquitination and K11-polyubiquitination. **s**. *In vitro* ubiquitination analysis for recombinant IRE1.KR as in **r** but using Me-Ub instead of Ub. Samples were analyzed by IRE1 IB. **t**. Identification of ubiquitination sites on recombinant IRE1.KR using UBE2D1 in the *in vitro* ubiquitination assay by mass-spectrometry. **u**. Identification of ubiquitination sites on recombinant IRE1.KR using UBE2D2 in the *in vitro* ubiquitination assay by mass-spectrometry.

Supplementary Figure 1 (continued)

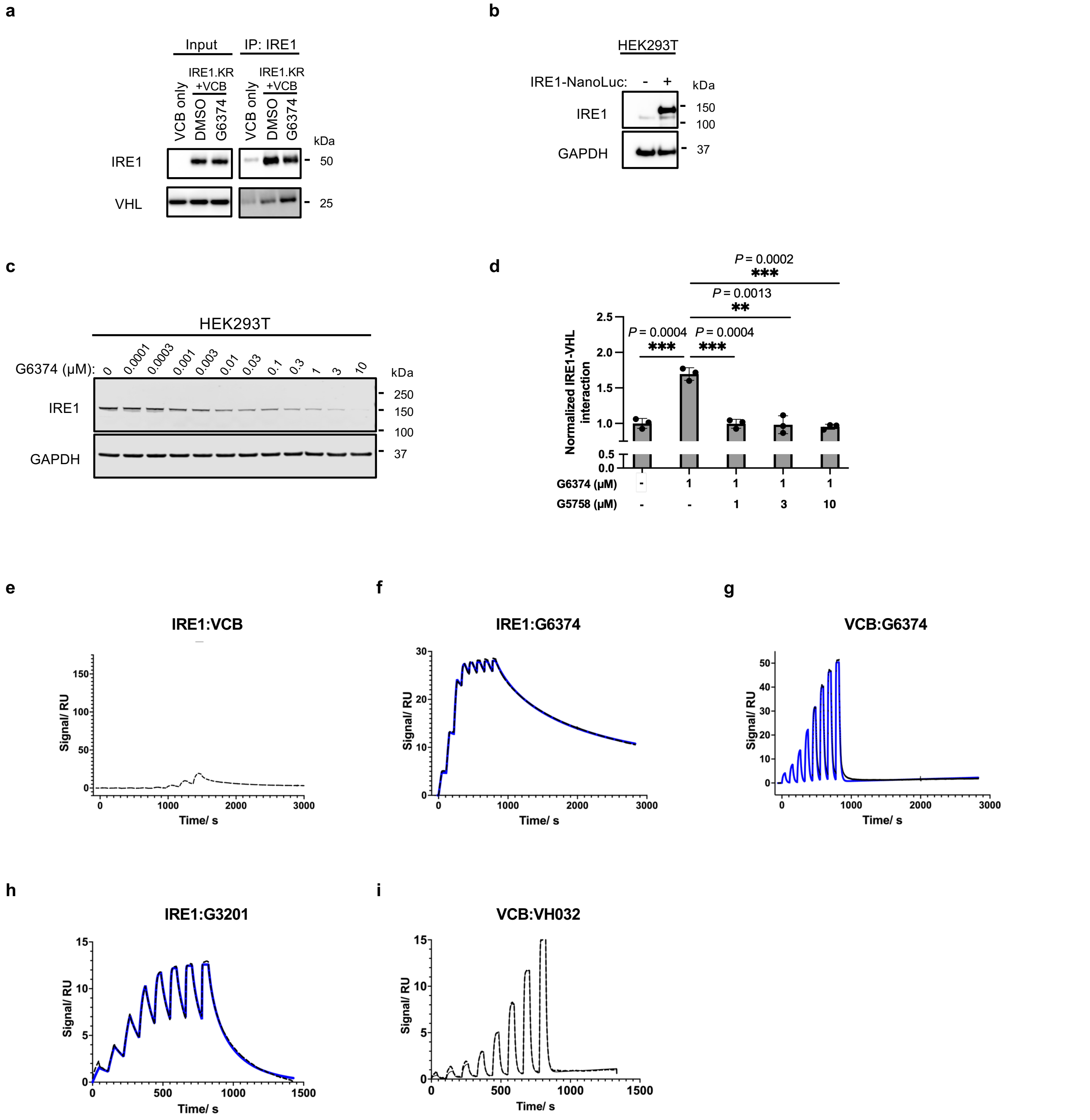

Supplementary Figure 2. G6374 promotes stable association between IRE1 and VHL. a. co-IP of recombinant VCB (50 nM) with recombinant IRE1.KR (50 nM) upon addition of DMSO or G6374 (100 nM). Samples were analyzed by IB for IRE1 and VHL. **b**. IB of IRE1 in HEK293T cells. The lower band in the IRE1 blot corresponds to endogenous IRE1 in HEK293T cells. The higher band corresponds to the ectopically expressed IRE1-NanoLuc protein. **c**. IB of IRE1 in HEK293T cells over-expressing IRE1-NanoLuc and treated with a titration of G6374 for 2 hr. **d**. NanoBRET assay with transfected IRE1 and VHL in HEK293T cells treated with 1 μM G6374 and a titration of the IRE1 kinase inhibitor, G5758, as a competitor. All NanoBRET signals were normalized to the "no G6374, no G5758" condition (n=3 replicates, mean ± SEM). **. $P < 0.01$; ***, $P < 0.001$. **e-i**. SRP sensorgrams for following binary complexes: IRE:VCB (**e**), IRE1:G6374 (**f**), VCB:G6374 (**g**), IRE1:G3201 (**h**) and VCB:VH032 (**i**).

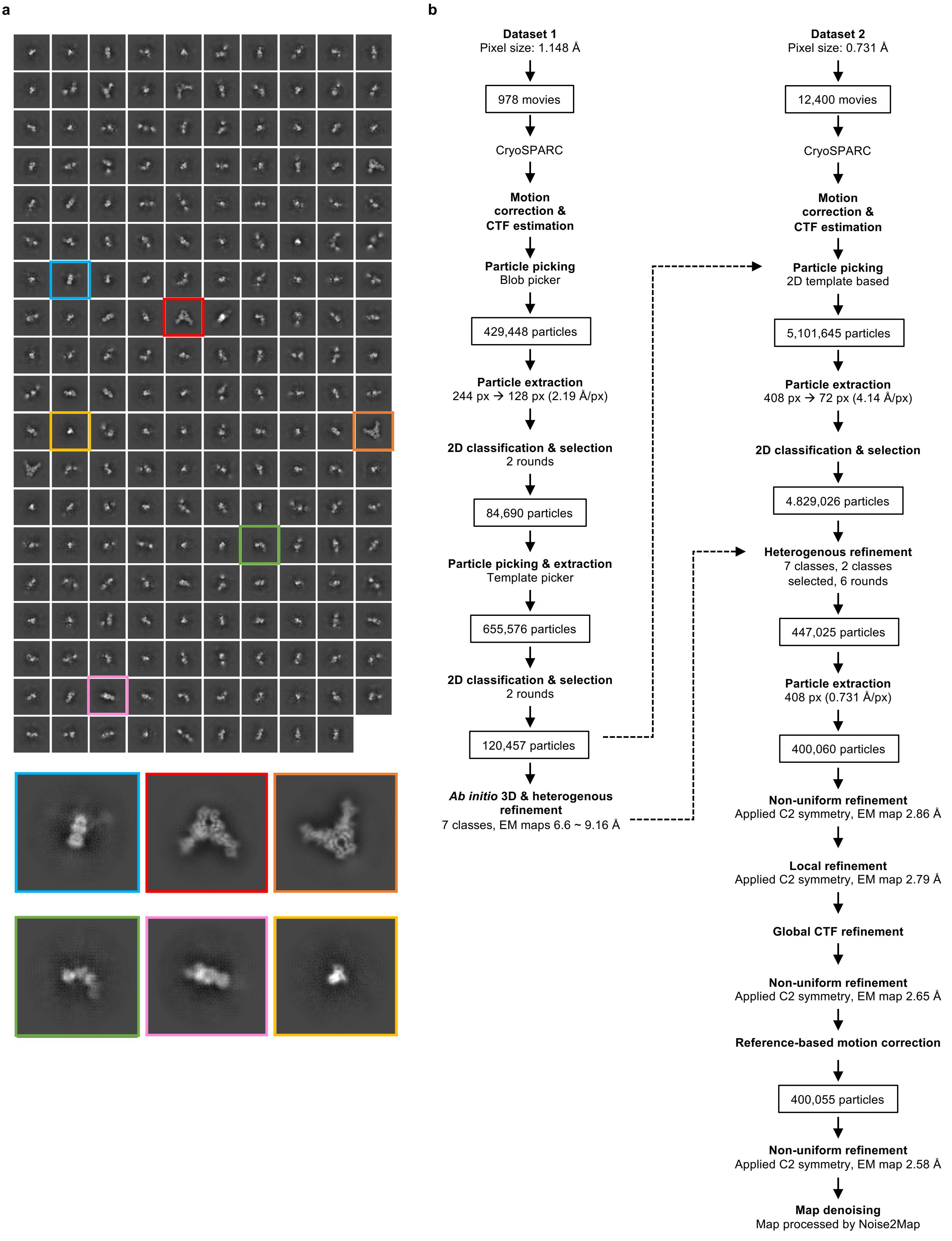

Supplementary Figure 3 (continued)

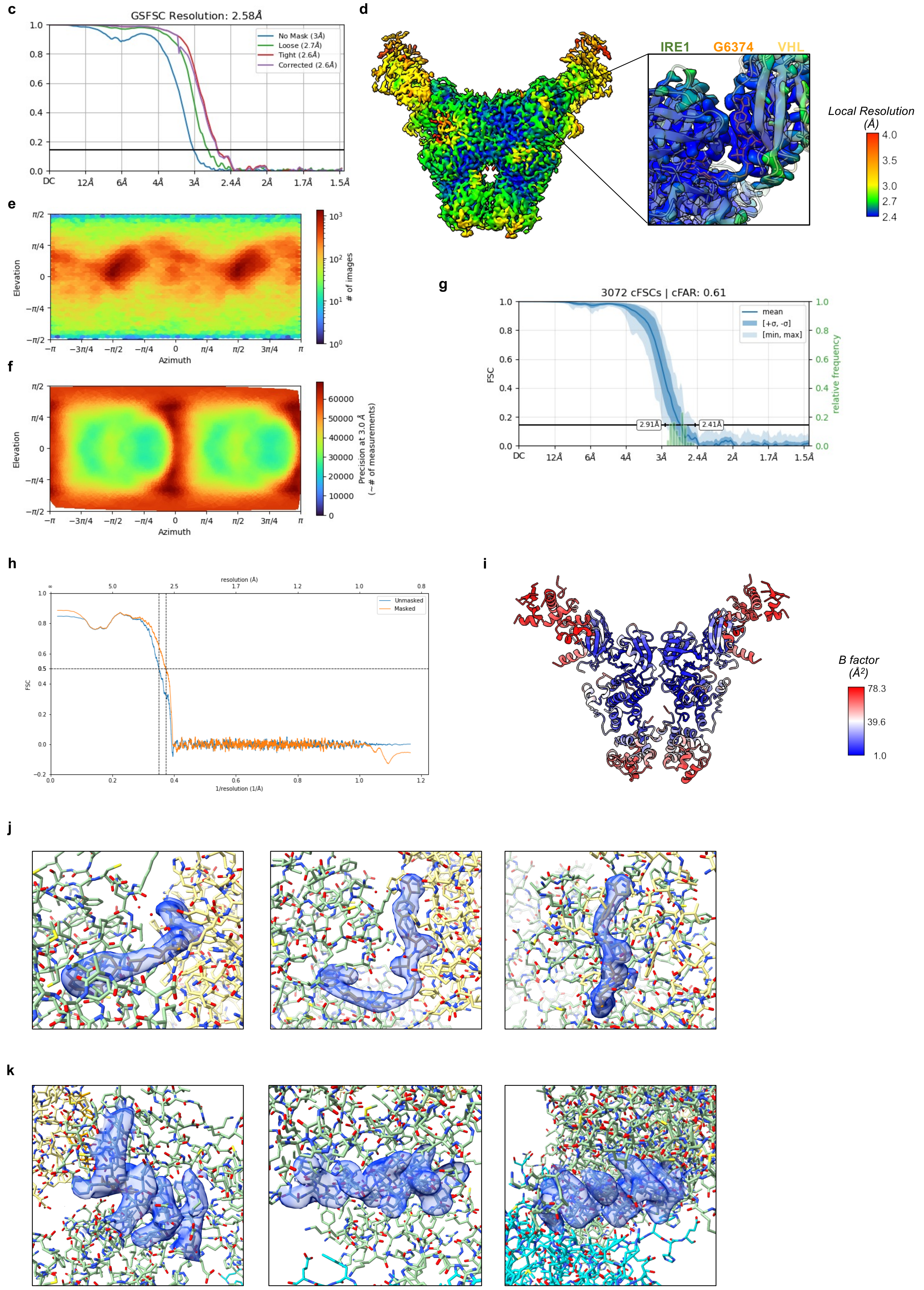

Supplementary Figure 3 (continued)

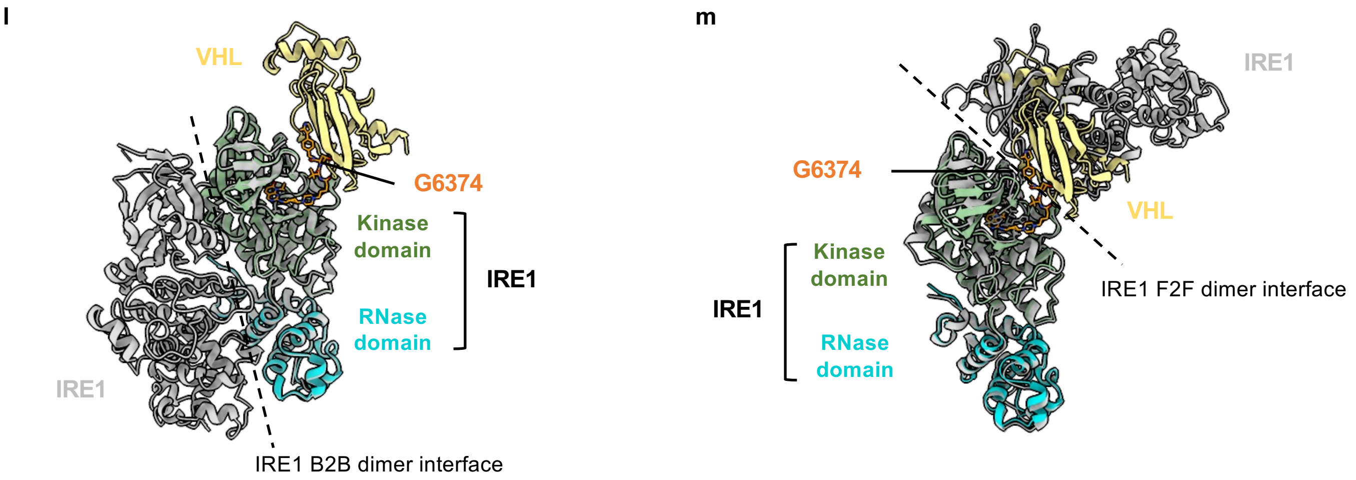

Supplementary Figure 3. **CryoEM structural analysis reveals the 3-D organization of the IRE1:G6374:VHL ternary complex. a**. Example 2-D class averages showing different orientations of the IRE1:G6374:VCB complex, with a few classes enlarged below. Box size = 298 Å. **b**. Flowchart of cryoEM image processing. **c**. Fourier Shell Correlation (FSC) of the 3-D reconstruction, indicating an estimated resolution of 2.6 Å. **d**. Local resolution estimated by two independently refined half maps, with a highlight of the IRE1:G6374:VHL interface. **e**. Direction distribution of particles. **f**. Precision directional distribution of particles. **g**. Conical Fourier Shell Correlation (cFSC) and conical FSC Area Ratio (cFAR) of the 3-D reconstruction, indicating a moderate level of orientation bias. **h**. Model-to-map FSC at 0.5, indicating an estimated resolution of 2.7 Å. **i**. Per-residue B-factors of the model. **j.** Representative angles of the model-to-map fit for G6374. **k.** Model-to-map fit for representative helices. **l**. Overlay of the IRE1back-to-back (B2B) dimer (PDB: 6W3B, colored in grey) to the IRE1:G6374:VHL complex. **m**. Overlay of the IRE1 face-to-face (F2F) dimer (PDB: 3P23, colored in grey) to the IRE1:G6374:VHL complex.

Supplementary Figure 3 (continued)

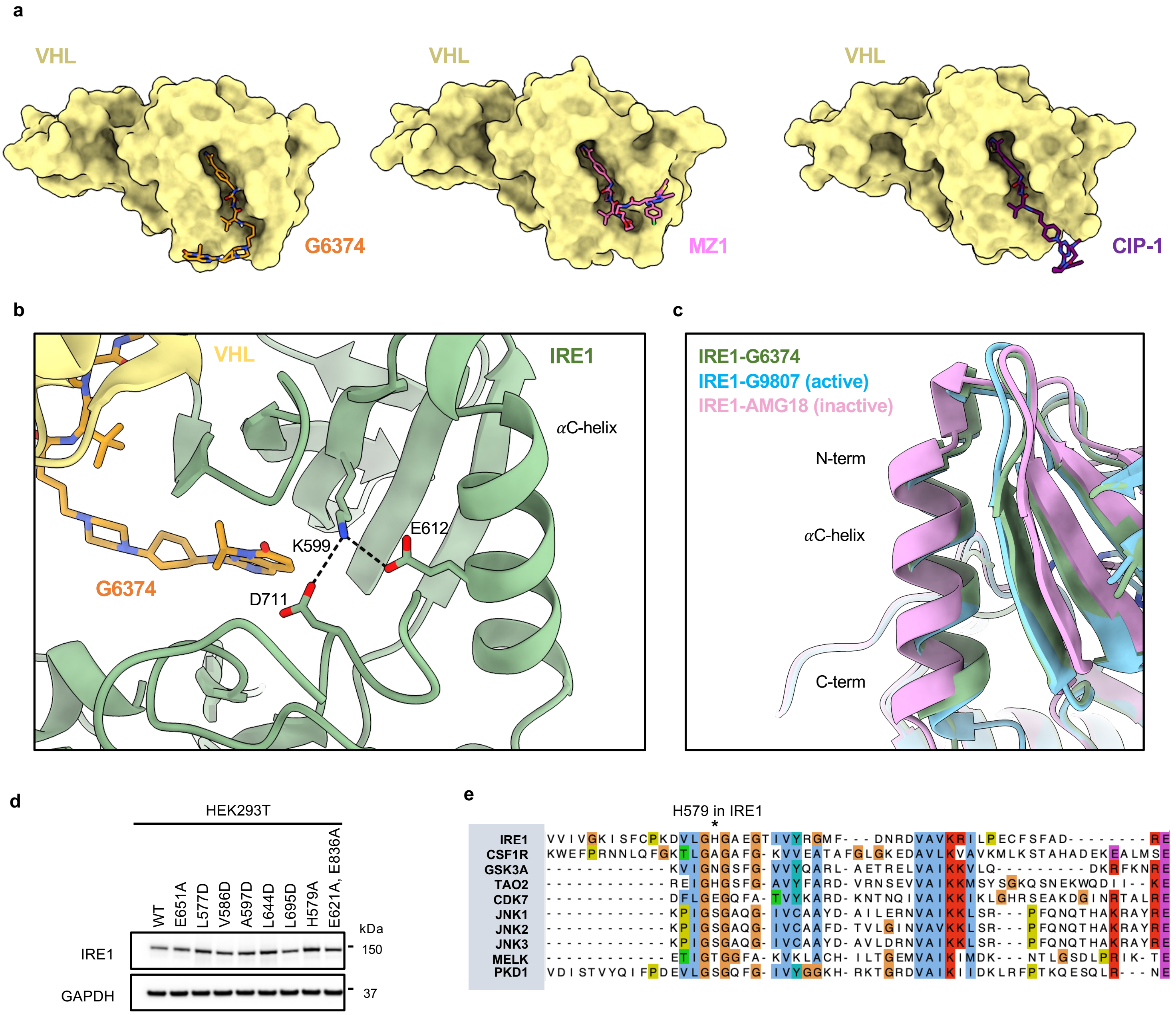

Supplementary Figure 4. Details of the VHL:G6374:IRE1 interface. a. Structures of VHL-G6374, VHL-MZ1 (PDB: 5T35) and VHL-CIP-1 (PDB: 8EWV) with the same orientation of the VHL ligand. The structures of VHL-MZ1 and VHL-CIP-1 are obtained from larger complexes in their original PDBs. **b**. Salt-bridge formation between K599 and E612 in the G6374-bound IRE1 structure. **c**. The αC-helices of superimposed IRE1-G6374, IRE1-G9807 (PDB: 6W3K; G9807[25] is an IRE1 activator) and IRE1-AMG18 (PDB: 6URC; AMG-18[73] is an IRE1 inhibitor). **d**. IB confirmation of IRE1-NanoLuc constructs with point mutations expressed in HEK293T cells. **e**. Conservation of H579 in kinases. Multiple sequence alignment of IRE1 near with major off-target kinase hits in the SelectScreen Kinase Profiling in **Fig. 1c** was performed with “Muscle with Defaults” in Jalview and visualized by Jalview (version 2.11.4.1). H579 in IRE1 is highlighted with an asterisk.

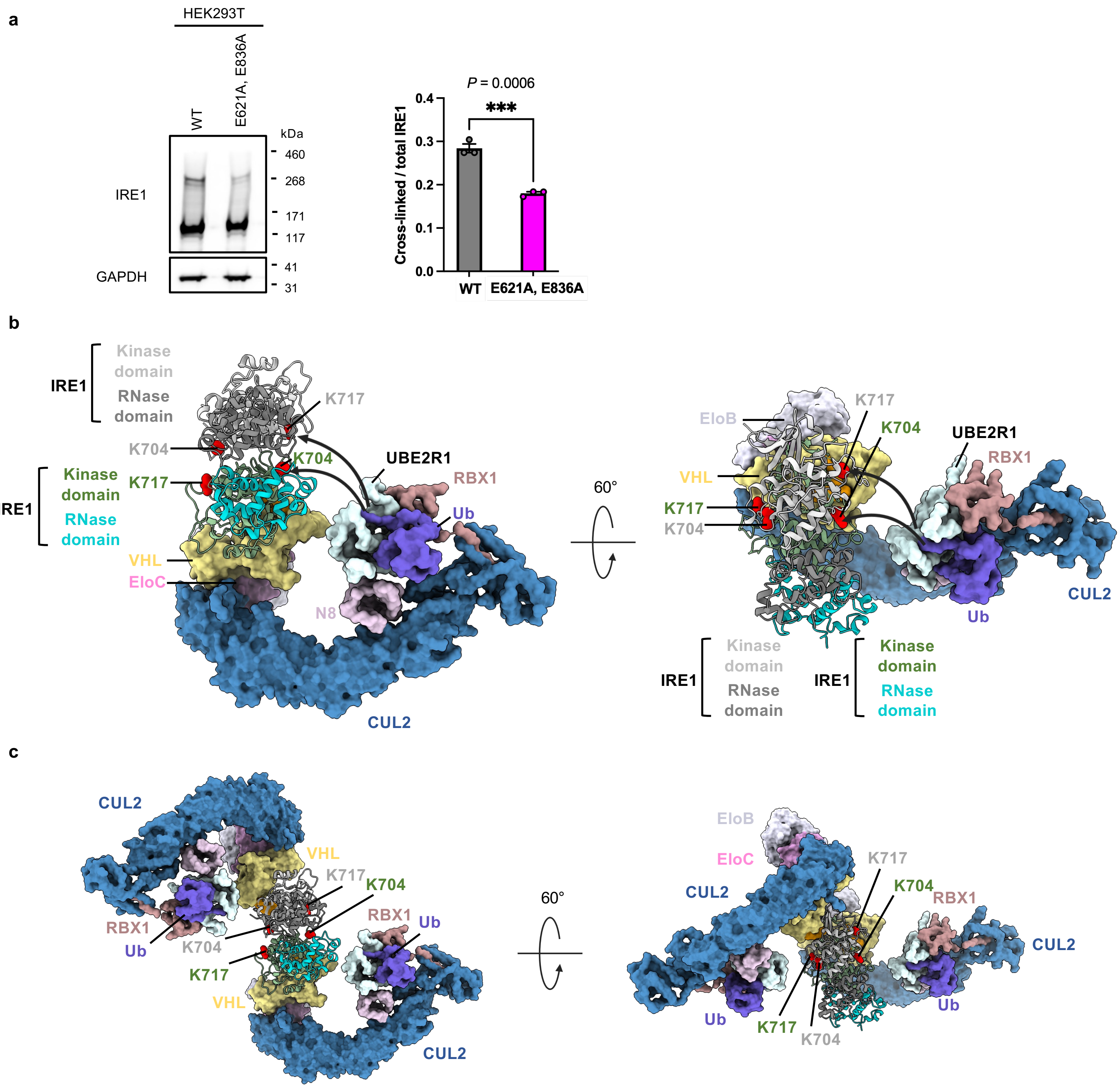

Supplementary Figure 5. G6374 can target monomeric IRE1 for degradation. a. IB of crosslinked WT or B2B dimer mutant (E621A, E836A) over-expressed in HEK293T cells using IRE1 antibody (left) with quantification of fraction of crosslinked IRE1 over total IRE1 (right; n=3 biological replicates, mean ± SEM). ***, P < 0.001. **b**. Model of IRE1 B2B dimer in complex with a full CRL2^{VHL} assembly (PDB: 8RX0). The arrows illustrate potential accessibility for Ub transfer by RBX1 to K704 on the immediately bound IRE1 protomer (green/cyan) as well as to K717 on the indirectly bound IRE1 protomer (grey/light grey) (Note: we used the IRE1.KR structure from PBD: 6W3B to model the complex because the side chain of K717 from our structure is less well resolved). **c**. Model of IRE1 B2B dimer in complex with 2 copies of full CRL2^{VHL} assembly (PDB: 8RX0).

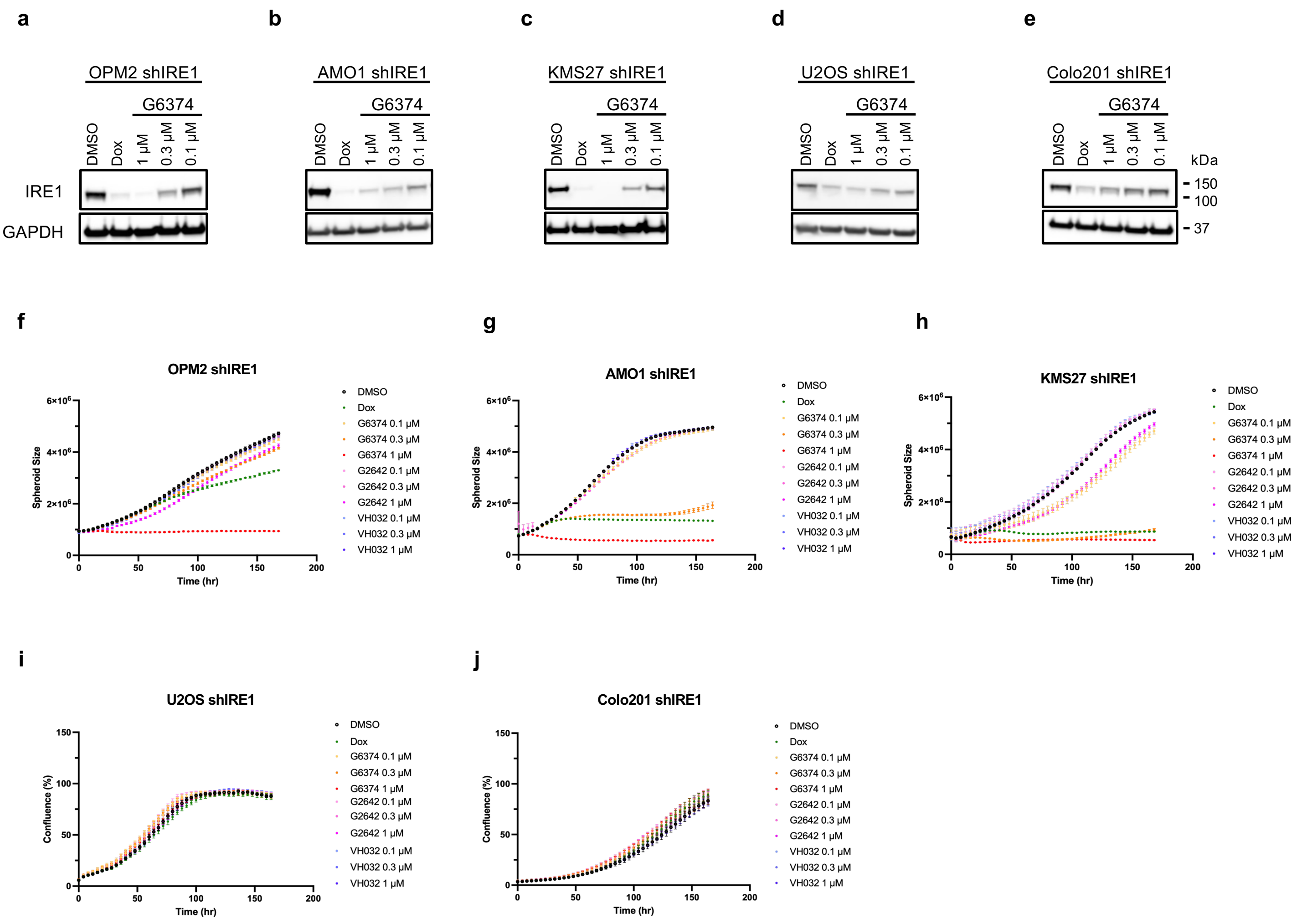

Supplementary Figure 6. **G6374 selectively inhibits IRE1-dependent cancer-cell proliferation regardless of the underlying mode of dependency. a-e.** IB validation of Dox-induced knockdown and G6374-induced degradation of endogenous IRE1 in OPM2 shIRE1 (**a**), AMO1 shIRE1 (**b**), KMS27 shIRE1 (**c**), U2OS shIRE1 (**d**) and Colo201 shIRE1 cells (**e**). **f-j.** Incucyte data (plotted with at least 3 replicates, mean ± SD) for OPM2 (**f**), AMO1 (**g**), KMS27 (**h**), U2OS (**i**) or Colo201 (**j**) cells expressing Dox-inducible shRNA against IRE1, treated with a titration of G6374, G2642 (the epimer control of G6374), or VH032 (the VHL ligand). Dox is used at 0.2 µg/ml as a control for IRE1 depletion.

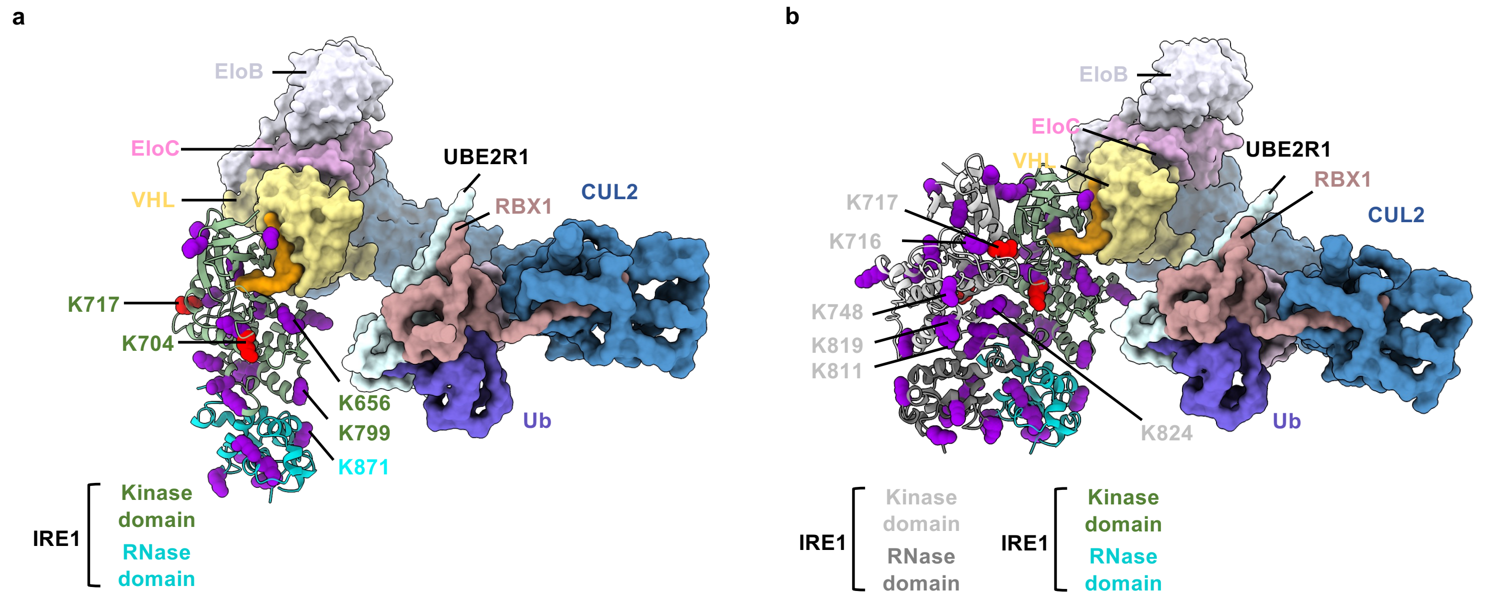

Supplementary Figure 7. Modeling IRE1 possible ubiquitination sites in the kinase-RNase domain. a, b. Model of either IRE1 monomer (**a**) or B2B dimer (**b**) in complex with a full CRL2^{VHL} assembly (PDB: 8RX0), with all lysine residues in IRE1.KR highlighted in purple, and K704 and K717 highlighted in red.

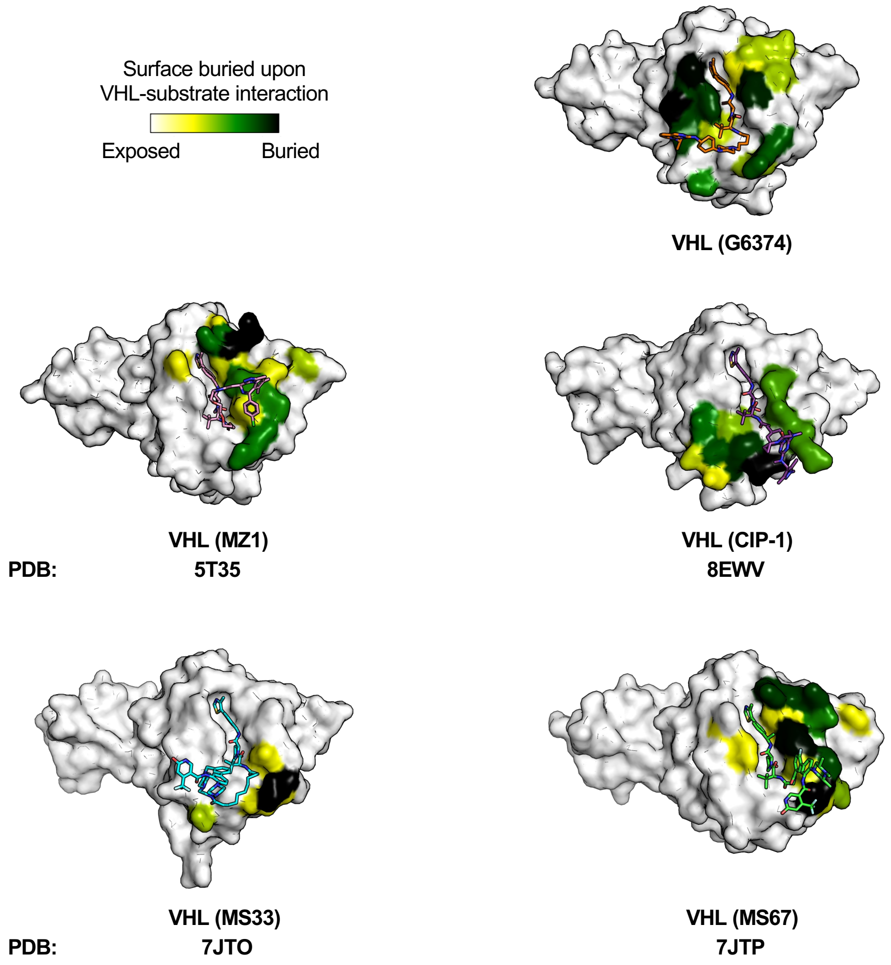

Supplementary Figure 8. **Comparison of VHL-based PROTAC structures reveals differences in VHL-substrate interaction interface.** Buried surface area on VHL resulting from VHL-IRE1 interaction (G6374), VHL-BRD4 interaction (MZ1, CIP-1), and VHL-WDR5 interaction (MS33, MS67), calculated by the PDBePISA server at the European Bioinformatics Institute (EBI), and visualized by PyMOL.

SUPPLEMENTARY TABLES

Supplementary Table 1. Data summary of surface plasmon resonance (SPR) and fluorescence polarization (FP).

	**Complex measured**	**k_a / $M^{-1}s^{-1}$**	**k_d / s^{-1}**	**K_d / nM**	**IC_{50} (nM)**	**Cooperativity alpha**
**SPR**	IRE1: VCB	N/A	N/A	>10000 (N=2)	N/A	N/A
	IRE1: G6374	4.95E+06 ± 2.85E+06 (N=2)	2.50E-03 ± 1.20E-03 (N=2)	0.56 ± 0.09 (N=2)	N/A	N/A
	VCB: G6374	5.14E+05 ± 2.24E+04 (N=3)	4.77E-02 ± 5.44E-03 (N=3)	90.23 ± 4.98 (N=3)	N/A	N/A
	IRE1: G3201	5.30E+06 (N=1)	1.42E-02 (N=1)	2.70 (N=1)	N/A	N/A
	VCB: VH032	5.50E+05 (N=1)	1.30E-01 (N=1)	232.00 (N=1)	N/A	N/A
**FP**	VCB: G6374	N/A	N/A	N/A	2476 ± 112 (N=3)	5 ± 0.15 (N=3)
	VCB: (G6374+IRE1)	N/A	N/A	N/A	492 ± 21 (N=3)	

G3201: the IRE1 ligand portion of G6374 (See **Fig. 1a**); VH032: the VHL ligand portion of G6374 (See **Fig. 1a**). Mean ± SEM with replicates (N) specified in brackets. The ternary complex cooperativity (alpha) was calculated as the ratio between IC_{50} of VCB: G6374 and VCB: (G6374 + IRE1).

Supplementary Table 2. CryoEM data collection, refinement and validation statistics.

	IRE1:G6374:VHL:EloC:EloB (EMDB-49119) (PDB 9N88)
**Data collection and processing**	
Magnification	165k
Voltage (kV)	300
Electron exposure (e–/Å^2)	45
Defocus range (μm)	-0.6 to -3.5
Pixel size (Å)	0.731
Symmetry imposed	C2
Initial particle images (no.)	5,101,645
Final particle images (no.)	400,055
Map resolution (Å)	2.58
FSC threshold	0.143
Map resolution range (Å)	2.4 to 4
**Refinement**	
Initial model used (PDB code)	6W3B, 5T35
Model resolution (Å)	2.6
FSC threshold	0.5
Model resolution range (Å)	2.4 to 4
Map sharpening *B* factor (Å^2)	-112.4
Model composition	
Non-hydrogen atoms	9766
Protein residues	1220
Ligands	2
B factors (Å^2)	
Protein	0.99/78.28/35.92
Ligand	0.98/16.40/7.28
R.m.s. deviations	
Bond lengths (Å)	0.004 (0)
Bond angles (°)	0.552 (0)
Validation	
MolProbity score	1.32
Clashscore	3.91
Poor rotamers (%)	1.55
Ramachandran plot	
Favored (%)	98.48
Allowed (%)	1.52
Disallowed (%)	0.00
EMRinger score	3.93

Supplementary Table 3. Comparison of buried surface areas in VHL-based PROTAC ternary complexes.

Buried surface areas in ternary complexes (Å^2)	VHL: G6374: IRE1	VHL: MZ1: BRD4	VHL: CIP-1: BRD4	VHL: AD122: WDR5	VHL: AD142: WDR5	VHL: AD157: WDR5	VHL: MS33: WDR5	VHL: MS67: WDR5
**Ligase : Target**	347	364	218	181	90	127	133	415
**Ligase : Ligand**	438	505	471	420	601	497	542	490
**Target : Ligand**	504	514	528	509	549	487	858	716
**Total**	1289	1383	1216	1110	1240	1111	1533	1621
**DC_{50} (nM)**	70	2~20	95	896	1557	722	260	3.7
**D_{max} (%)**	95	98	91	38	28	21	71	94
**PDB ID**	9N88	5T35	8EWV	7Q2J	8BB5	8BB4	7JTO	7JTP

Buried surface areas are calculated by commands: “measure buriedarea (/A & protein) withAtoms2 (/B & protein)” for protein:protein, and “measure buriedarea /A withAtoms2 :LIG” for protein:ligand in ChimeraX (version 1.7.1). DC_{50} and D_{max} values were obtained from references 32, 51, 57, 63, 64.

SUPPLEMENTARY METHODS

Chemical synthesis of G6374

8-isopropyl-2-(((1r,4r)-4-(piperazin-1-yl)cyclohexyl)amino)pyrido[2,3-d]pyrimidin-7(8H)-one hydrochloride (IRE1-Int)

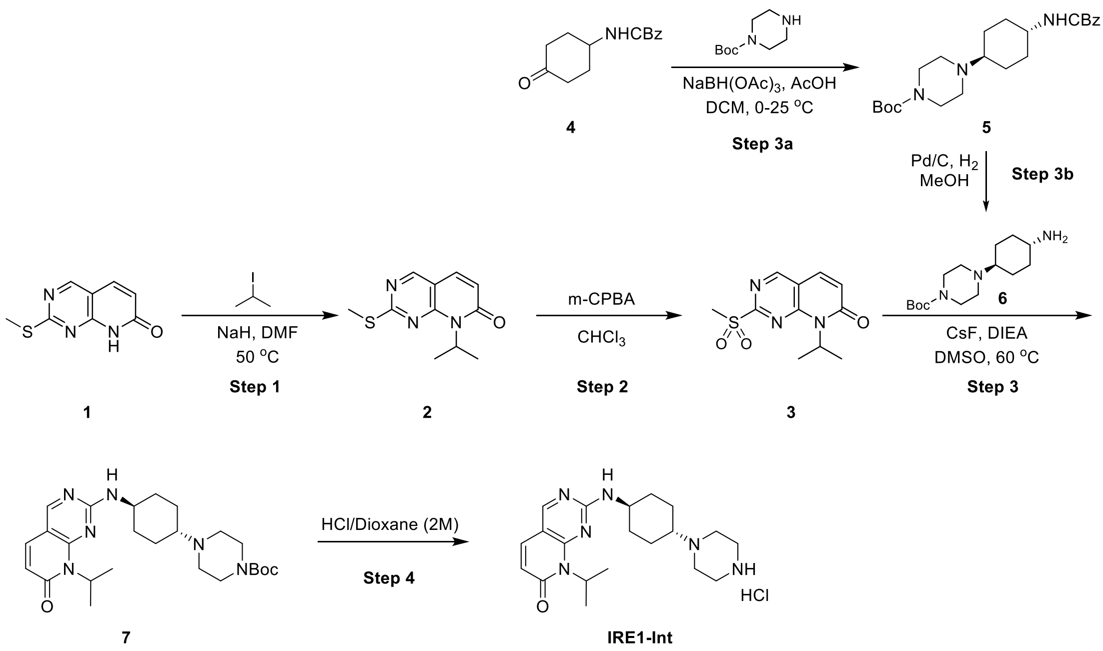

Step 1: 8-isopropyl-2-(methylthio)pyrido[2,3-d]pyrimidin-7(8H)-one (2)

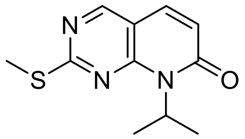

To a solution of NaH (3.11 g, 77.6 mmol, 60% purity) in DMF (70.0 mL) was added compound **1** (10.0 g, 51.7 mmol). The mixture was stirred at 50 °C for 0.5 hr. Then added 2-iodopropane (10.5 g, 62.1 mmol, 6.21 mL) at 25 °C and stirred at 50 °C for 0.5 hr. The reaction mixture was quenched by addition H_2O (400 mL) at 0°C, and extracted with Ethyl acetate (200 mL x 3). The combined organic layers were washed with brine (50.0 mL), dried over Na_2SO_4, filtered and concentrated under reduced pressure to give a residue. The residue was purified by column chromatography (SiO_2, Petroleum ether/Ethyl acetate=1/0 to 0/1). Compound **2** (8.00 g, 34.0 mmol, 65.7% yield) was obtained as a white solid.

Step 2: 8-isopropyl-2-(methylsulfonyl)pyrido[2,3-d]pyrimidin-7(8H)-one (3)

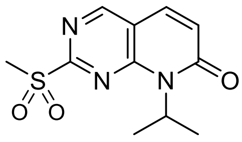

To a solution of compound **2** (8.00 g, 34.0 mmol) in $CHCl_3$ (60.0 mL) was added m-CPBA (20.7 g, 102 mmol, 85% purity). The mixture was stirred at 25 °C for 12 hrs. The reaction mixture was quenched by addition Na_2SO_3 (100 mL), and then diluted with H_2O (50.0 mL) and extracted with $CHCl_3$ (20.0 mL x 3). The combined organic layers were washed with brine 50.0 mL, dried over Na_2SO_4, filtered and concentrated under reduced pressure to give a residue. Compound **3** (7.00 g, 26.2 mmol, 77.0% yield) was obtained as a yellow solid. ^{1}H NMR (400 MHz, DMSO-d_6) δ 9.26 (s, 1H), 8.06 (d, *J* = 8.0 Hz, 1H), 6.87 (d, *J* = 8.0 Hz, 1H), 5.65 (m, 1H), 3.46 (s, 3H), 1.56 (d, *J* = 8.0 Hz, 6H).

Step 3a: tert-butyl 4-((1r,4r)-4-((2-oxo-2-phenyl-1λ^2-ethyl)amino)cyclohexyl)piperazine-1-carboxylate (5)

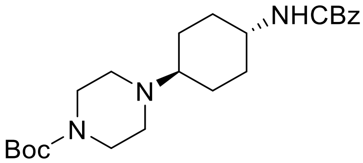

To a solution of compound **4** (50.0 g, 202 mmol) in DCM (350 mL) was added tert-butyl piperazine-1-carboxylate;hydrochloride (45.0 g, 202 mmol), HOAc (4.98 g, 82.9 mmol, 4.74 mL) at 0-10°C, then was added $NaBH(OAc)_3$ (128 g, 606 mmol) at 0°C, then the mixture was stirred at 25°C for 2 hours. The reaction mixture was added saturation $NaHCO_3$ aqueous solution to make pH = 7. The resulting mixture was extracted with DCM (300 mL, 200 mL. 150 mL) and combined. The combined organic phase was washed with brine (80.0 mL) and separated, dried over Na_2SO_4, filtered and the filtrate was concentrated in vacuum to give a crude product. The crude product was purified by flash chromatography on a silica gel eluted with petroleum ether/EtOAc (from 100/1 to 1/1). Then the product was purified by prep-HPLC (column: Welch Xtimate C18 250*70mm#10um; mobile phase: [water(10mM NH4HCO3)-ACN];B%: 45%-60%,20min). Compound **5** (30.0 g, 71.8 mmol, 35.5% yield) was obtained as a white solid. ^{1}H NMR (400 MHz, $CDCl_3$) δ 7.35-7.30 (m, 5 H), 5.07 (s, 2

H), 4.61-4.59 (m, 1 H), 3.41-3.39 (m, 5 H), 2.47 (s, 4 H), 2.25 (s, 1 H), 2.09-2.06 (m, 2 H), 1.89-1.86 (m, 2 H), 1.44 (s, 9 H), 1.35-1.32 (m, 2 H), 1.14-1.11 (m, 2 H).

Step 3b: tert-butyl 4-((1r,4r)-4-aminocyclohexyl)piperazine-1-carboxylate (6)

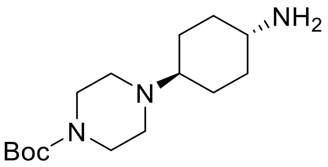

To a solution of compound **5** (15.0 g, 35.9 mmol) in MeOH (90.0 mL) was added Pd/C (1.50 g, 35.9 mmol, 10% purity) under N_2. The suspension was degassed under vacuum and purged with H_2 (35.9 mmol) several times. The mixture was stirred under H_2 (50.0 psi) at 25°C for 12 hours. The reaction mixture was filtered and the filter was concentrated. The crude product would be directly used in the next step without purification. Compound **6** (19.7 g, crude) was obtained as a white solid.

Step 4: tert-butyl 4-((1r,4r)-4-((8-isopropyl-7-oxo-7,8-dihydropyrido[2,3-d]pyrimidin-2-yl)amino)cyclohexyl)piperazine-1-carboxylate (7)

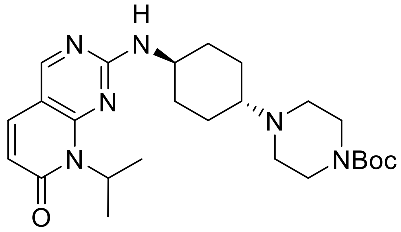

A mixture of compound **3** (2.00 g, 7.48 mmol) , compound **6** (2.33 g, 8.23 mmol) , CsF (3.41 g, 22.4 mmol, 827 uL) , DIEA (2.90 g, 22.4 mmol, 3.91 mL) in DMSO (20.0 mL) was degassed and purged with N_2 for 3 times, and then the mixture was stirred at 60 °C for 12 hrs under N_2 atmosphere. The reaction mixture was diluted with H_2O 100 mL and extracted with Ethyl acetate (50.0 mL * 3). The combined organic layers were washed with brine (20.0 mL), dried over Na_2SO_4, filtered and concentrated under reduced pressure to give a residue. The residue was purified by prep-HPLC (column: Phenomenex Titank C18 Bulk 250*100mm 10u; mobile phase: [water (0.05%NH3H2O+10mM NH4HCO3)-ACN]; B%: 40%-70%, 20min). Compound **7**

(2.02 g, 4.29 mmol, 57.3% yield, 100% purity) was obtained as a yellow solid. LCMS (ESI): m/z 471.3 $(M+H)^+$. ^{1}H NMR (400 MHz, MeOD) δ 8.48 (s, 1H), 7.64 (d, *J* = 9.2 Hz, 1H), 6.27- 6.25 (m, 1H), 5.89-5.74 (m, 1H), 3.82 (m, 1H), 3.44-3.30 (m, 4H), 2.60-2.57 (m, 4H), 2.40 (m, 1H), 2.16-2.02 (m, 4H), 1.61-1.50 (m, 6H), 1.49-1.38 (m, 13H).

Step 5: 8-isopropyl-2-(((1*r*,4*r*)-4-(piperazin-1-yl)cyclohexyl)amino)pyrido[2,3-*d*]pyrimidin-7(8*H*)-one hydrochloride (IRE1-Int)

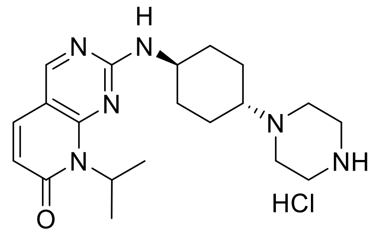

To a solution of compound **5** (400.0 mg, 0.85 mmol) in dioxane (5 mL) was added 2M HCl in dioxane (10 mL) at 25 °C. Then the reaction mixture was stirred at 25 °C for 2 h. After that, the reaction mixture was concentrated under reduced pressure to afford the title compound (345.0 mg, 99%) as a yellow solid. LCMS (ESI): m/z 371.2 $(M+H)^+$.

2-(((1r,4r)-4-(dimethylamino)cyclohexyl)amino)-8-isopropylpyrido[2,3-d]pyrimidin-7(8H)-one (G1167)

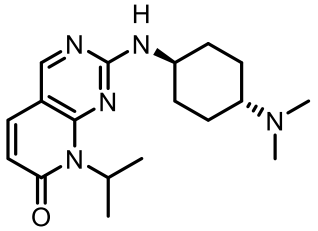

G1167 was prepared in a method analogous to IRE1-Int above. HRMS (ESI+): m/z calcd for $C_{18}H_{27}N_5O$ (M+H)+ 330.2288., found 330.2280. Additional signals present due to the presence of rotamers, only major peaks are reported: 1H NMR (400 MHz, DMSO) δ 8.52 (s, 1H), 7.63 (d, J = 9.2 Hz, 1H), 6.19 (d, J = 9.2 Hz, 1H), 5.62 (s, 1H), 3.67 (s, 1H), 2.17 (s, 6H), 2.14 – 2.07 (m, 1H), 2.03 – 1.90 (m, 2H), 1.86 (d, J = 12.2 Hz, 2H), 1.53 (d, J = 6.9 Hz, 6H), 1.40 – 1.18 (m, 5H).

G1167 HRMS & LCMS:

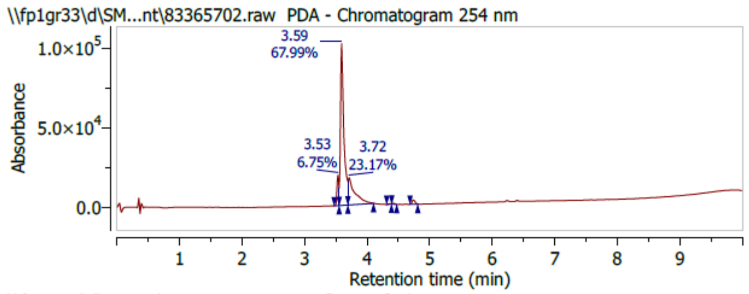

DAD, Sig=254 (>=1.5%)

RT	Area	Area %	MS+
3.53	188543.62	6.75	330.23
3.59	1898035.50	67.99	330.23
3.72	646670.88	23.17	330.23
4.36	4104.00	0.15	330.23
4.42	8194.00	0.29	330.19
4.74	45911.00	1.64	344.21

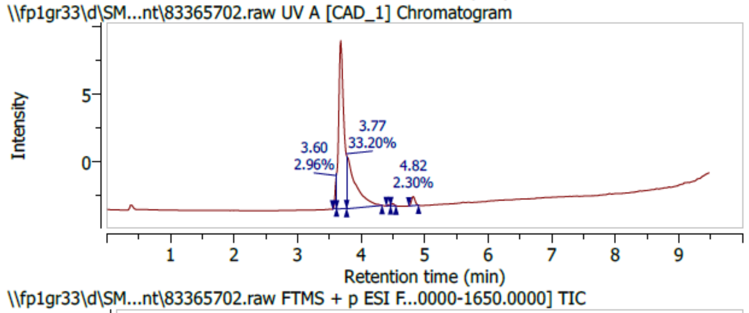

CAD_1 (>=5%)

RT	Area	Area %	MS+
3.60	66876440794.39	2.96	243.12
3.68	1378446973405.41	60.91	330.23
3.77	751332866369.01	33.20	330.23
4.44	4651043288.26	0.21	330.23
4.50	9837505461.41	0.43	330.19
4.82	52007845555.56	2.30	344.21

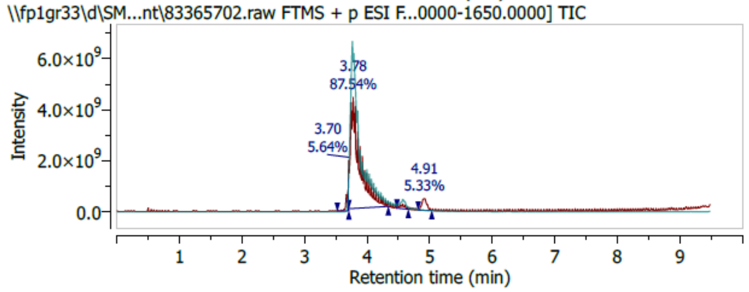

TIC (>=2%)

RT	Area	Area %	MS+
3.70	6977699496.92	5.64	316.21
3.78	108388636661.86	87.54	330.23
4.57	1860307520.00	1.50	330.23
4.91	6595218246.00	5.33	344.21

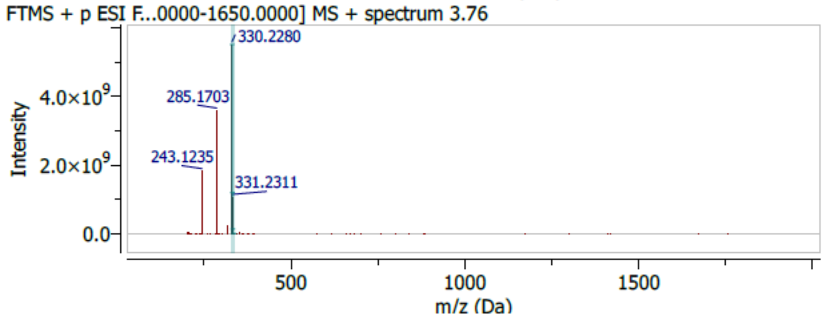

FTMS + p ESI F...0000-1650.0000] MS + spectrum 3.72

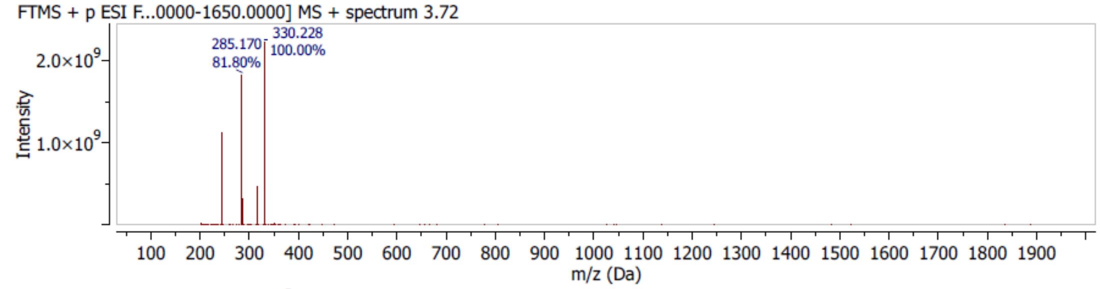

FTMS + p ESI F...0000-1650.0000] MS + spectrum 3.78

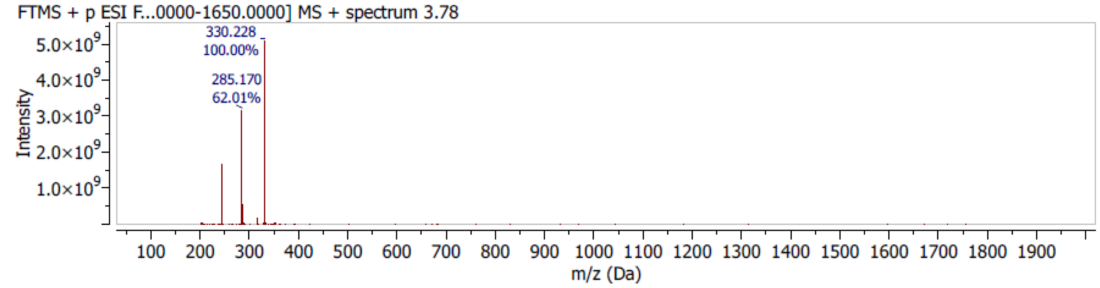

FTMS + p ESI F...0000-1650.0000] MS + spectrum 3.90

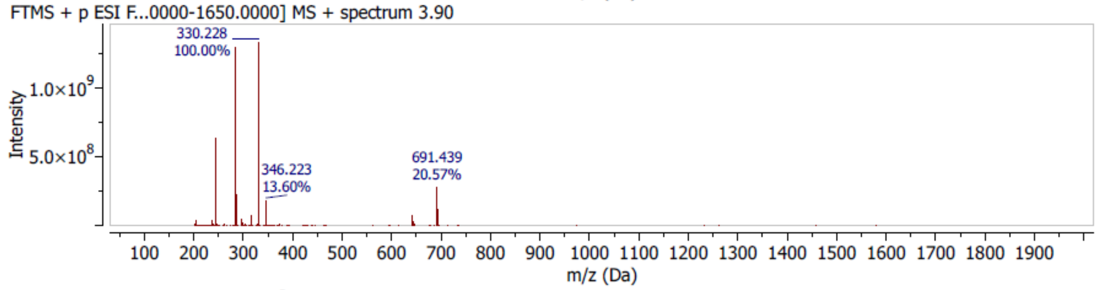

FTMS + p ESI F...0000-1650.0000] MS + spectrum 4.56

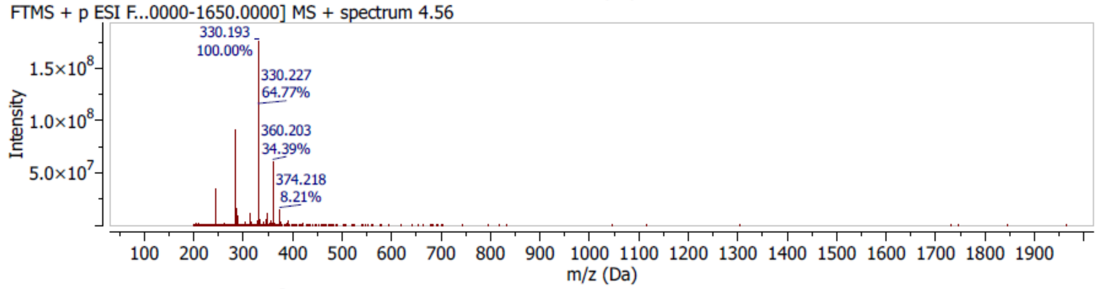

FTMS + p ESI F...0000-1650.0000] MS + spectrum 4.61

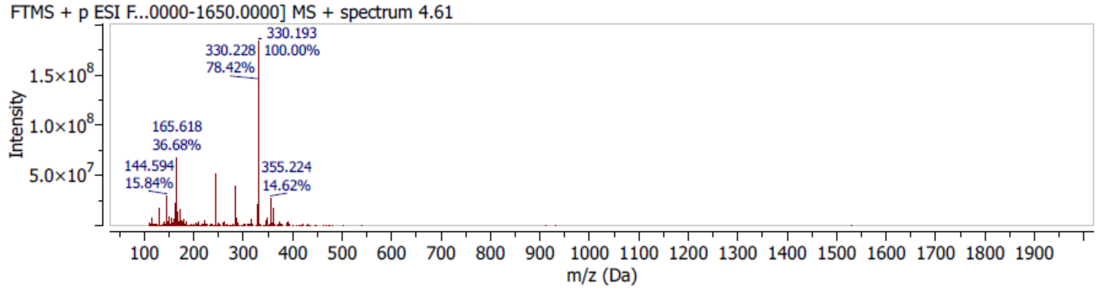

FTMS + p ESI F...0000-1650.0000] MS + spectrum 4.92

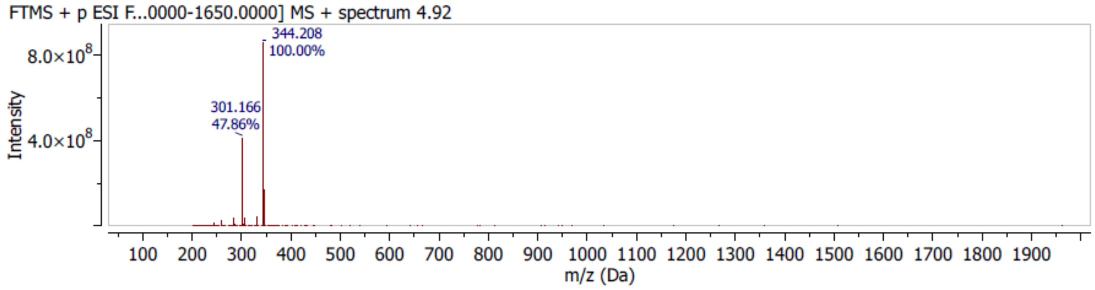

G1167 ^{1}H NMR:

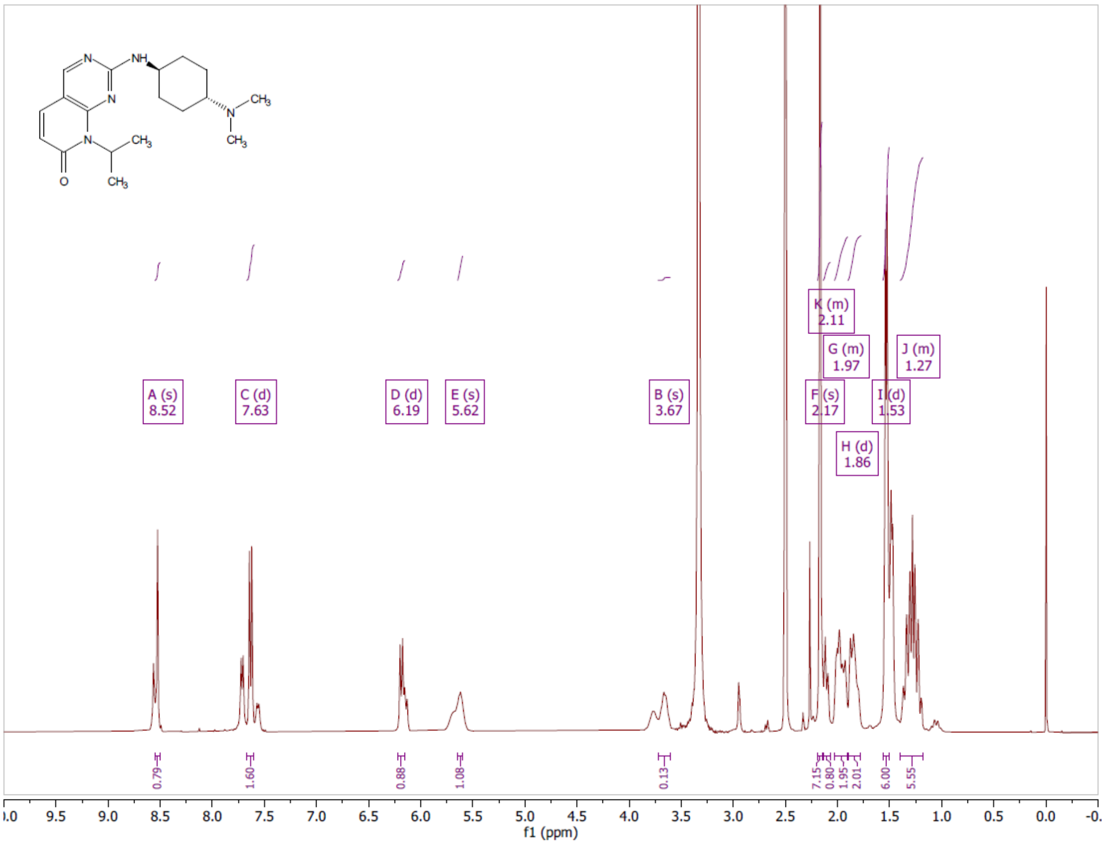

(2*S*,4*R*)-4-hydroxy-1-((*S*)-2-(5-(4-((1*r*,4*S*)-4-((8-isopropyl-7-oxo-7,8-dihydropyrido[2,3-*d*]pyrimidin-2-yl)amino)cyclohexyl)piperazin-1-yl)pentanamido)-3,3-dimethylbutanoyl)-*N*-(4-(4-methylthiazol-5-yl)benzyl)pyrrolidine-2-carboxamide (G6374)

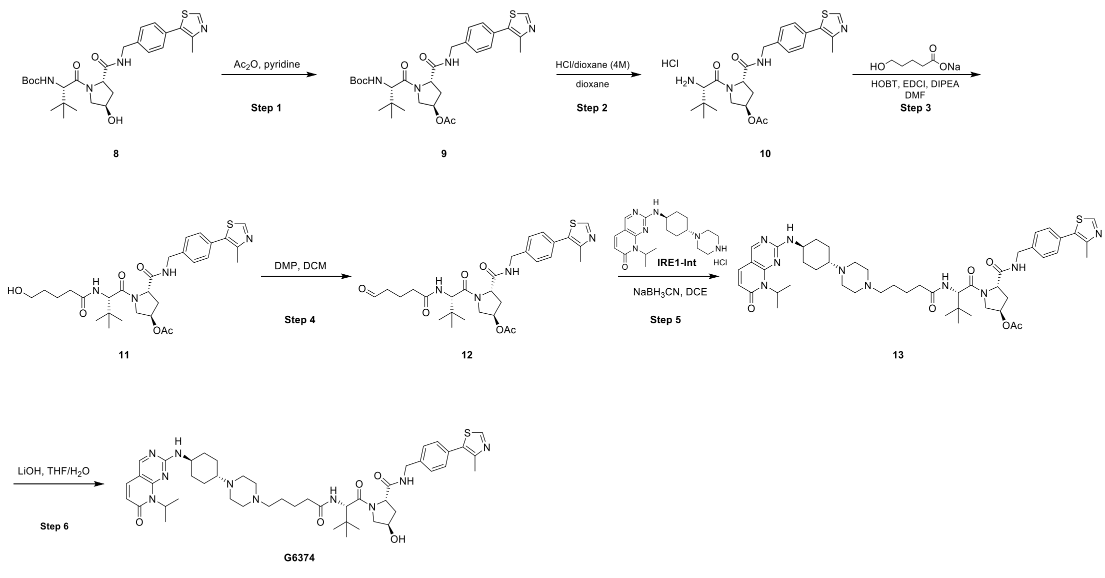

Step 1: (3R,5S)-1-((S)-2-((tert-butoxycarbonyl)amino)-3,3-dimethylbutanoyl)-5-((4-(4-methylthiazol-5-yl)benzyl)carbamoyl)pyrrolidin-3-yl acetate (9)

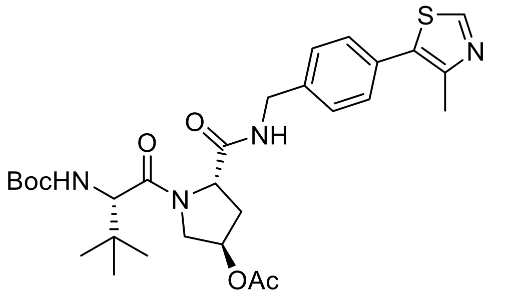

To a solution of compound **8** (50.0 g, 94.2 mmol) in pyridine (350 mL) was added acetyl acetate (48.1 g, 471 mmol, 44.1 mL), then the mixture was stirred at 25°C for 2 hours. The reaction mixture was added a saturated of $NaHCO_3$ solution to make PH = 8 and concentrated in vacuum to remove the pyridine, then extracted with EtOAc (200 mL x 3), the combined organic phase was washed with brine (5.00 mL), dried with anhydrous Na_2SO_4, filtered and concentrated in vacuum to afford compound **9** (110 g, crude) as a crude yellow oil.

Step 2: (3R,5S)-1-((S)-2-amino-3,3-dimethylbutanoyl)-5-((4-(4-methylthiazol-5-yl)benzyl)carbamoyl)pyrrolidin-3-yl acetate hydrochloride (10)

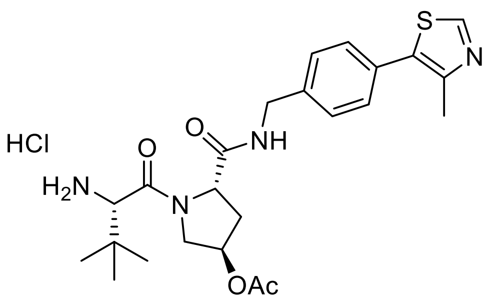

To a solution of compound **9** (108 g, 188 mmol) in dioxane (300 mL) was added HCl/dioxane (4 M, 432 mL), then the mixture was stirred at 25°C for 2 hours. The reaction mixture was concentrated in vacuum to give the product. The crude product was triturated with MTBE at 25°C for 30 min to afford compound **10** (105 g, 175 mmol, 93.0% yield, 85% purity, HCl) as a yellow solid.

Step 3: (3R,5S)-1-((S)-2-(5-hydroxypentanamido)-3,3-dimethylbutanoyl)-5-((4-(4-methylthiazol-5-yl)benzyl)carbamoyl)pyrrolidin-3-yl acetate (11)

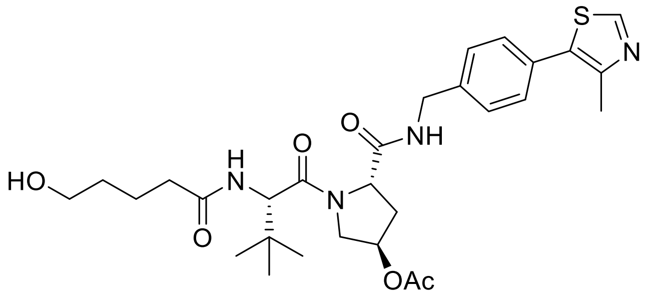

To a solution of compound **10** (8.50 g, 16.7 mmol) and 5-hydroxypentanoyloxysodium (2.81 g, 20.0 mmol) in DMF (60.0 mL) was added DIEA (6.47 g, 50.1 mmol, 8.72 mL), HOBt (2.26 g, 16.7 mmol) and EDCI (4.80 g, 25.0 mmol), the mixture was stirred at 25°C for 2 hours. The reaction mixture was concentrated under vacuum to give the crude product. The crude product was purified by reversed-phase HPLC(0.1% MeOH or 0.1% NH_3HCO_3 condition) to afford compound **11** (6.68 g, 11.4 mmol, 68.3% yield, 97.9% purity) as a white solid. LCMS (ESI): m/z 573.1 $(M+H)^+$. ^1H NMR (400 MHz, MeOD) δ 8.88 (s, 1H), 7.88 (d, *J* = 8.8 Hz, 1H), 7.48-7.40 (m, 4H), 5.37 (s, 1H), 4.88-4.51 (m, 3H), 4.35 (d, *J* = 15.2 Hz, 1H), 4.16 (d, *J* = 8.0 Hz, 1H), 3.95-3.3.87 (m, 1H), 3.55 (t, *J* = 6.4 Hz, 2H), 2.49 (s, 3H), 2.31-2.24 (m, 4H), 2.05 (s, 3H), 1.67-1.65 (m, 2H), 1.56-1.54 (m, 2H), 1.07 (s, 9H).

Step 4: (3*R*,5*S*)-1-((*S*)-3,3-dimethyl-2-(5-oxopentanamido)butanoyl)-5-((4-(4-methylthiazol-5-yl)benzyl)carbamoyl)pyrrolidin-3-yl acetate (12)

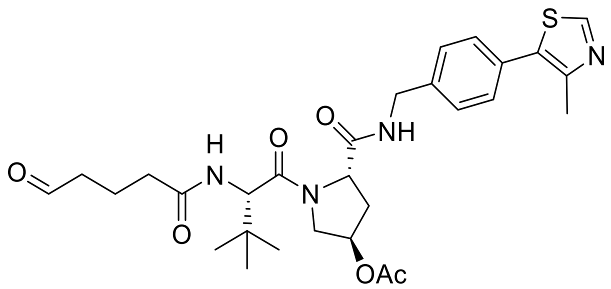

To a solution of compound **11** (400.0 mg, 0.7 mmol) in DCM (6 mL) was added DMP (355.0 mg, 0.84 mmol) at 25 °C. The resulting reaction mixture was stirred at 40 °C for 2 h. After that, the reaction mixture was cooled to room temperature and quenched by the dropwise addition of saturated aqueous Na_2SO_3 (5 mL) solution. Then the mixture was diluted with H_2O (60 mL), extracted with EtOAc (40 mL × 2). The combined organic layer was washed by brine (30 mL × 3), dried over anhydrous sodium sulfate, filtered and the filtrate was concentrated under reduced pressure to afford the title compound (350 mg, 88%) as a yellow oil. LCMS (ESI): m/z 593.1 $(M+Na)^+$.

Step 5: (3*R*,5*S*)-1-((*S*)-2-(5-(4-((1*r*,4*S*)-4-((8-isopropyl-7-oxo-7,8-dihydropyrido[2,3-*d*]pyrimidin-2-yl)amino)cyclohexyl)piperazin-1-yl)pentanamido)-3,3-dimethylbutanoyl)-5-((4-(4-methylthiazol-5-yl)benzyl)carbamoyl)pyrrolidin-3-yl acetate (13)

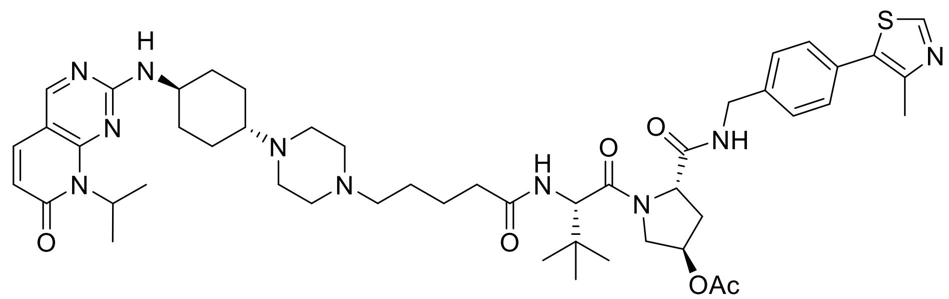

To a stirred solution of compound **12** (350.0 mg, 0.61 mmol) in DCE (5 mL) was added 8-isopropyl-2-(((1*r*,4*r*)-4-(piperazin-1-yl)cyclohexyl)amino)pyrido[2,3-

d]pyrimidin-7(8*H*)-one hydrochloride (**IRE1-Int**) (312.0 mg, 0.77 mmol) and $NaBH_3CN$ (260.0 mg, 1.23 mmol) at 25 °C. The resulting reaction mixture was stirred at 25 °C for 16 h. Then the reaction was quenched by the dropwise addition of saturated aqueous NH_4Cl (20 mL) solution. The mixture was diluted with H_2O (20 mL), extracted with EtOAc (20 mL × 2). The combined organic layer was washed by brine (30 mL × 3), dried over anhydrous sodium sulfate and concentrated under vacuum. The residue was purified by column chromatography on silica gel eluting with 0 - 10% MeOH in DCM to afford the title compound (120.0 mg, 21%) as a yellow solid. LCMS (ESI): m/z 925.5 $(M+H)^+$.

Step 6: (2*S*,4*R*)-4-hydroxy-1-((*S*)-2-(5-(4-((1*r*,4*S*)-4-((8-isopropyl-7-oxo-7,8-dihydropyrido[2,3-*d*]pyrimidin-2-yl)amino)cyclohexyl)piperazin-1-yl)pentanamido)-3,3-dimethylbutanoyl)-*N*-(4-(4-methylthiazol-5-yl)benzyl)pyrrolidine-2-carboxamide (G6374)

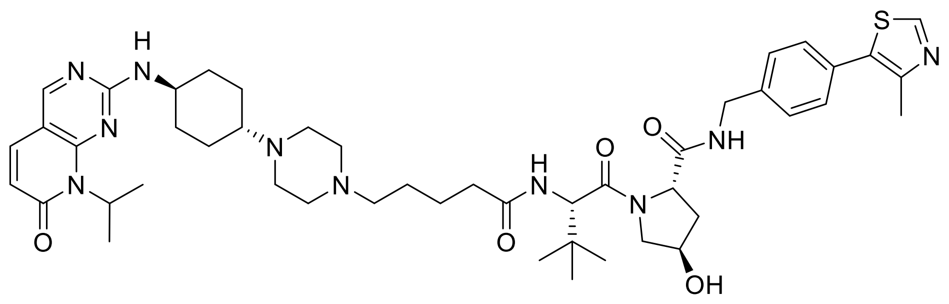

To a mixture of compound **13** (120.0 mg, 0.13 mmol) in water (1 mL) and THF (3 mL) was added LiOH monohydrate (27.3 mg, 0.65 mmol) at 25 °C. The resulting reaction mixture was stirred at 25 °C for 3 h. Then the reaction mixture was purified by pre-HPLC (acetonitrile 58-88/0.225% FA in water) to afford the title compound (60.0 mg, 51%) as a white solid. HRMS (ESI+): m/z calcd for $C_{47}H_{66}N_{10}O_5S$ (M+H)+ 883.5011, found 883.5006. 1H NMR (400 MHz, DMSO) δ 8.98 (s, 1H), 8.55 (dd, *J* = 13.2, 6.7 Hz, 2H), 8.19 (s, 1H), 7.85 (d, *J* = 9.3 Hz, 1H), 7.72 (d, *J* = 7.7 Hz, 1H), 7.64 (d, *J* = 9.3 Hz, 1H), 7.40 (q, *J* = 8.2 Hz, 4H), 6.17 (dd, *J* = 17.3, 9.2 Hz, 1H), 4.54 (d, *J* = 9.3 Hz, 1H), 4.44 (q, *J* = 7.4 Hz, 2H), 4.35 (s, 1H), 4.24 (dd, *J* = 16.5, 6.9 Hz, 1H), 3.72 – 3.60 (m, 3H), 2.45 (s, 3H), 2.35 (s, 4H), 2.32 – 1.81 (m, 13H), 1.51 (dd, *J* = 19.4, 6.9 Hz, 8H), 1.42 - 1.24 (m, 6H), 0.94 (s, 9H). ^{13}C NMR (101 MHz, DMSO) *additional

signals present due to the presence of rotamers δ 172.06, 171.96, 169.72, 163.54, 163.03, 160.45*, 160.36, 159.17, 159.07*, 155.66, 151.47, 147.72, 139.52, 136.68, 136.55*, 131.17, 129.64, 128.64, 127.43, 116.62*, 115.82, 105.23*, 104.81, 68.86, 62.04*, 61.96, 58.70, 57.51, 56.36, 56.30, 53.15, 50.13, 49.58*, 48.60, 48.46*, 44.02, 41.65, 37.96, 35.21, 34.71, 31.40*, 31.07, 27.00, 26.81*, 26.39, 25.78, 23.39, 19.43, 19.13*, 15.95.

G6374 HRMS & LCMS:

\\fp1gr33\d\SM...nt\86450688.raw PDA - Chromatogram 254 nm

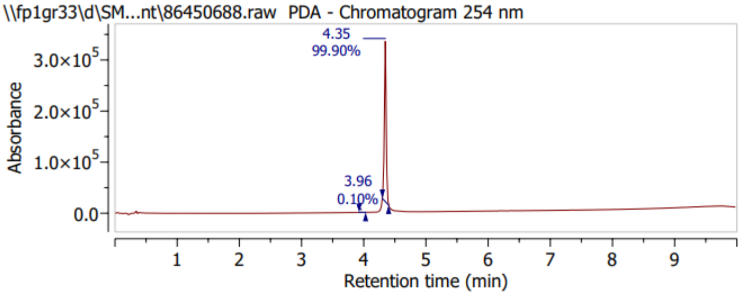

DAD, Sig=254 (>=1.5%)

RT	Area	Area %	MS+
3.96	3509.35	0.10	219.06
4.35	3340028.50	99.90	442.25

\\fp1gr33\d\SM...nt\86450688.raw CAD_1 Chromatogram 0 Da

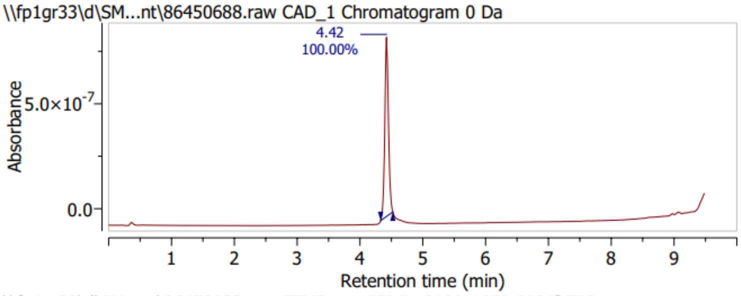

CAD_1 (>=5%)

RT	Area	Area %	MS+
4.42	67750.55	100.00	442.25

\\fp1gr33\d\SM...nt\86450688.raw FTMS + p ESI F...0000-1650.0000] TIC

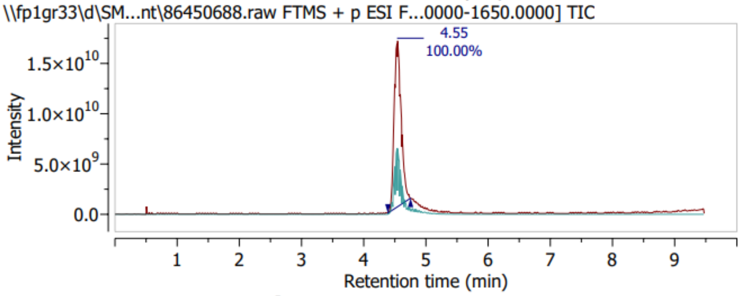

TIC (>=2%)

RT	Area	Area %	MS+
4.55	356743213105.00	100.00	442.25

FTMS + p ESI F...0000-1650.0000] MS + spectrum 4.53

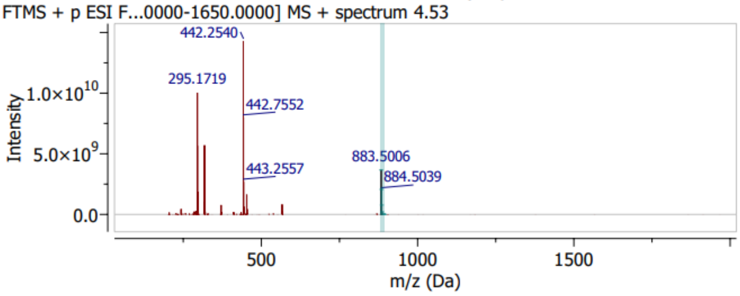

\\fp1gr33\d\SM...nt\86450688.raw Injection 1 FTMS + p ESI F...0000-1650.0000] MS + spectrum 4.15

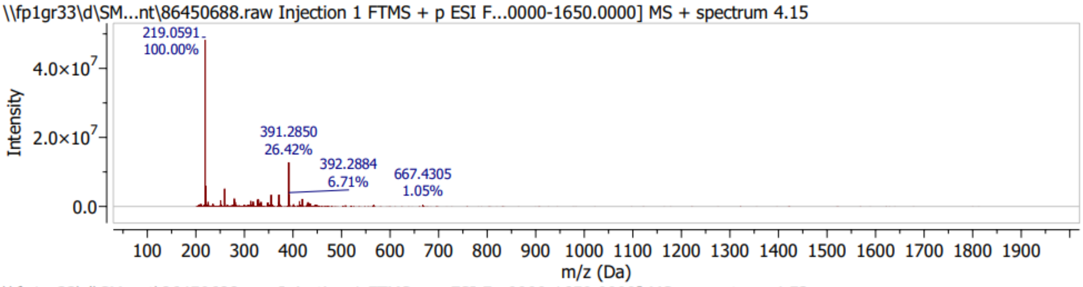

\\fp1gr33\d\SM...nt\86450688.raw Injection 1 FTMS + p ESI F...0000-1650.0000] MS + spectrum 4.53

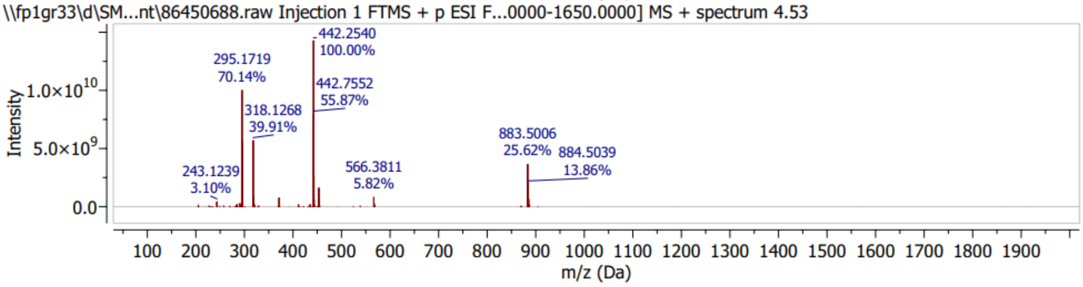

G6374 ^{1}H NMR:

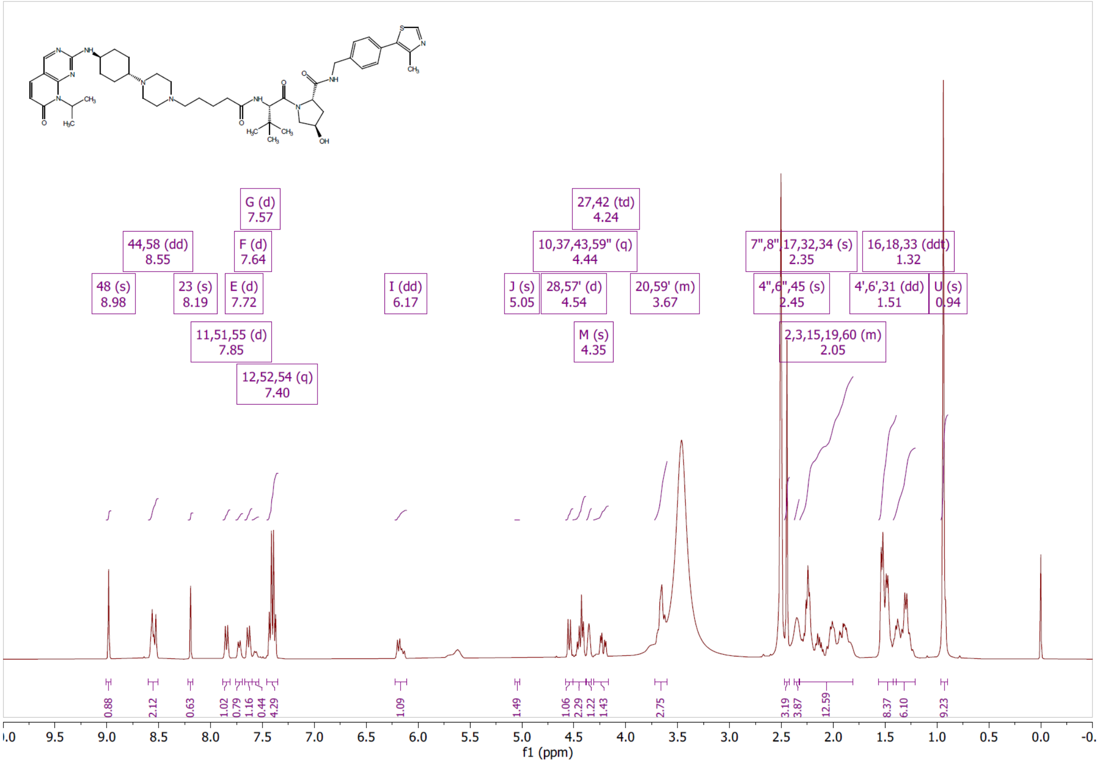

G6374 ^{13}C NMR:

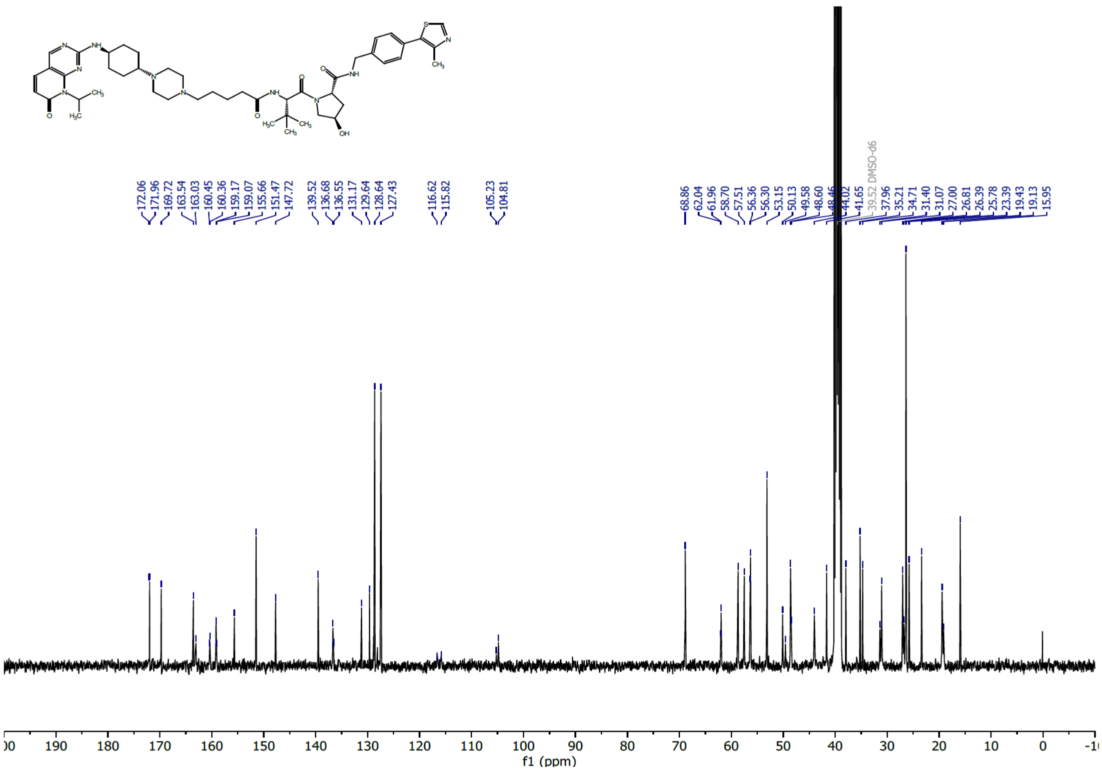

(2*S*,4*S*)-4-hydroxy-1-((*S*)-2-(5-(4-((1*r*,4*S*)-4-((8-isopropyl-7-oxo-7,8-dihydropyrido[2,3-d]pyrimidin-2-yl)amino)cyclohexyl)piperazin-1-yl)pentanamido)-3,3-dimethylbutanoyl)-N-(4-(4-methylthiazol-5-yl)benzyl)pyrrolidine-2-carboxamide (G2642)

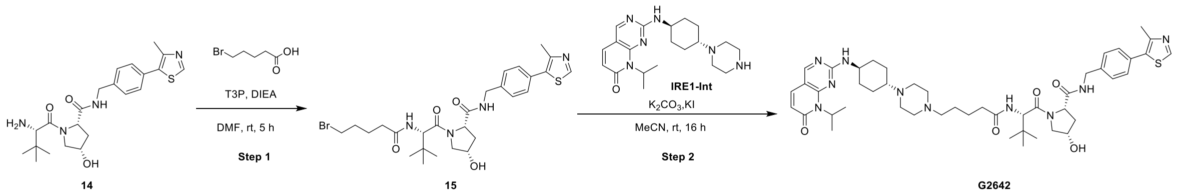

Step 1: (2*S*,4*S*)-1-((*S*)-2-(5-bromopentanamido)-3,3-dimethylbutanoyl)-4-hydroxy-N-(4-(4-methylthiazol-5-yl)benzyl)pyrrolidine-2-carboxamide

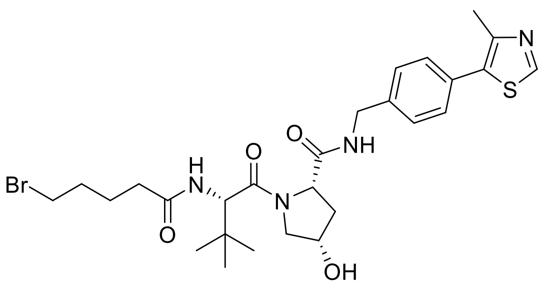

To a mixture of 5-bromopentanoic acid (0.09 mL, 0.93 mmol), compound **14** (200.0 mg, 0.46 mmol) and *N*,*N*-diisopropylethylamine (0.4 mL, 2.32 mmol) in DCM (3 mL) was added 2,4,6-tripropyl-1,3,5,2,4,6-trioxatriphosphorinane-2,4,6-trioxide (0.55 mL, 0.93 mmol) at 0 °C. The resulting reaction mixture was stirred at 25 °C for 5 h. After that, the reaction mixture was diluted with water (15 mL), extracted with DCM (25 mL× 2). The combined organic layer was washed with brine (20 mL), dried over anhydrous sodium sulfate, filtered, and the filtrate was concentrated under reduced pressure. The residue was purified by column chromatography on silica gel eluting with 0-1% MeOH in DCM to afford the title compound (200 mg, 73%) as a white solid. ^{1}H NMR (400 MHz, DMSO-d_6) δ 8.99 (s, 1H), 8.64 (t, J = 6.0 Hz, 1H), 7.91 (d, J = 8.8 Hz, 1H), 7.42 - 7.37 (m, 4H), 5.44 (d, J = 7.2 Hz, 1H), 4.49 - 4.36 (m, 3H), 4.30 - 4.18 (m, 2H), 3.94 - 3.91 (m, 1H), 3.53 (t, J = 6.4 Hz, 2H), 3.45 - 3.42 (m, 1H), 2.44 (s, 3H), 2.35 - 2.32 (m, 2H), 2.20 - 2.11 (m, 1H), 1.79 - 1.69 (m, 3H), 1.64 - 1.53 (m, 2H), 0.95 (s, 9H).

Step 2: (2*S*,4*S*)-4-hydroxy-1-((*S*)-2-(5-(4-((1*r*,4*S*)-4-((8-isopropyl-7-oxo-7,8-dihydropyrido[2,3-d]pyrimidin-2-yl)amino)cyclohexyl)piperazin-1-

yl)pentanamido)-3,3-dimethylbutanoyl)-N-(4-(4-methylthiazol-5-yl)benzyl)pyrrolidine-2-carboxamide (G2642)

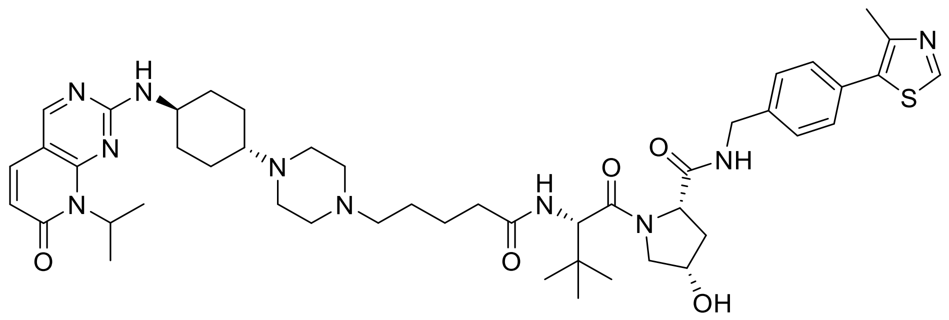

To a solution of compound **15** (34 mg, 0.09 mmol) in acetonitrile (3 mL) were added potassium carbonate (23 mg, 0.17 mmol), *rac*-(2*S*,4*S*)-4-hydroxy-N-[[4-(4-methylthiazol-5-yl)phenyl]methyl]-1-[*rac*-(2*S*)-2-(5-bromopentanoylamino)-3,3-dimethyl-butanoyl]pyrrolidine-2-carboxamide (50.0mg, 0.08mmol) and potassium iodide (16.6 mg, 0.1 mmol) at 25 °C. The resulting reaction mixture was then stirred at 25 °C for 16 h. After that, the reaction mixture was purified by reverse phase chromatography (acetonitrile 1 - 28% / 0.2% formic acid in water) to afford the title compound (12.5 mg, 16%) as a white soild. LCMS (ESI): *m/z* 883.4 $(M+H)^+$. ^{1}H NMR (400 MHz, DMSO-d_6) δ 8.98 (s, 1H), 8.66 - 8.60 (m, 1H), 8.58 - 8.51 (m, 1H), 8.19 (s, 1H), 7.85 (d, *J* = 8.8 Hz, 1H), 7.75 - 7.55 (m, 2H), 7.43 - 7.37 (m, 4H), 6.23 - 6.10 (m, 1H), 5.63 - 5.61 (m, 1H), 4.49 - 4.41 (m, 2H), 4.39 - 4.32 (m, 1H), 4.29 - 4.18 (m, 2H), 3.97 - 3.91 (m, 1H), 3.84 - 3.59 (m, 4H), 2.46 - 2.42 (m, 4H), 2.40 - 2.28 (m, 5H), 2.27 - 2.22 (m, 5H), 2.16 - 2.09 (m, 1H), 2.03 - 1.91 (m, 2H), 1.90 - 1.79 (m, 2H), 1.78 - 1.70 (m, 1H), 1.56 - 1.44 (m, 8H), 1.43 - 1.19 (m, 7H), 0.95 (s, 9H).

G2642 HPLC:

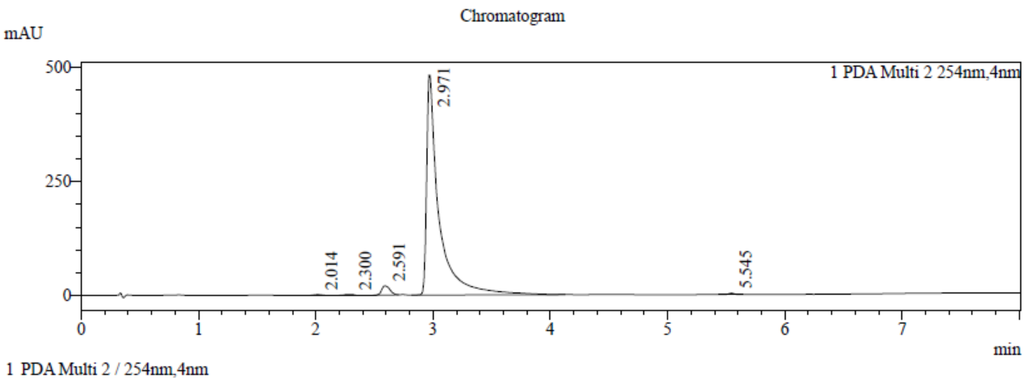

1 PDA Multi 2 / 254nm,4nm

Integration Result

Peak Table

PDA Ch2 254nm

Peak#	Ret. Time	Height	Height%	USP Width	Area	Area%
1	2.014	1712	0.335	0.153	8535	0.231
2	2.300	2517	0.493	0.161	13851	0.375
3	2.591	20538	4.019	0.149	100690	2.725
4	2.971	482922	94.494	0.163	3560968	96.360
5	5.545	3371	0.660	0.103	11457	0.310

G2642 LCMS:

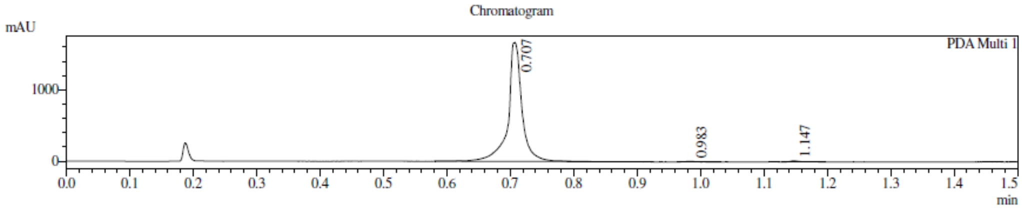

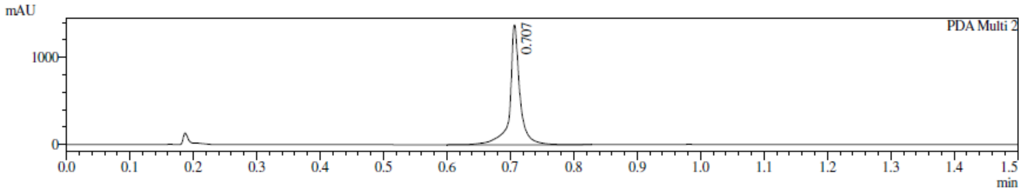

1 PDA Multi 1 / 220nm 4nm
2 PDA Multi 2 / 254nm 4nm

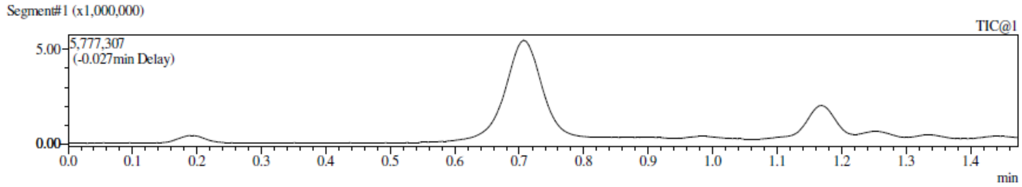

Integration Result

PDA Ch1 220nm 4nm

Peak#	Ret. Time	Height	Height %	USP Width	Area	Area %
1	0.707	1660318	98.936	0.029	2561517	99.194
2	0.983	5494	0.327	0.028	6715	0.260
3	1.147	12359	0.736	0.023	14090	0.546

PDA Ch2 254nm 4nm

Peak#	Ret. Time	Height	Height %	USP Width	Area	Area %
1	0.707	1344835	100.000	0.022	1522771	100.000

MS Spectrum Graph

RetTime: 0.707 DataFile: D:\DATA\2021\2110\211020\GNEWX-1937-608-1A1.lcd

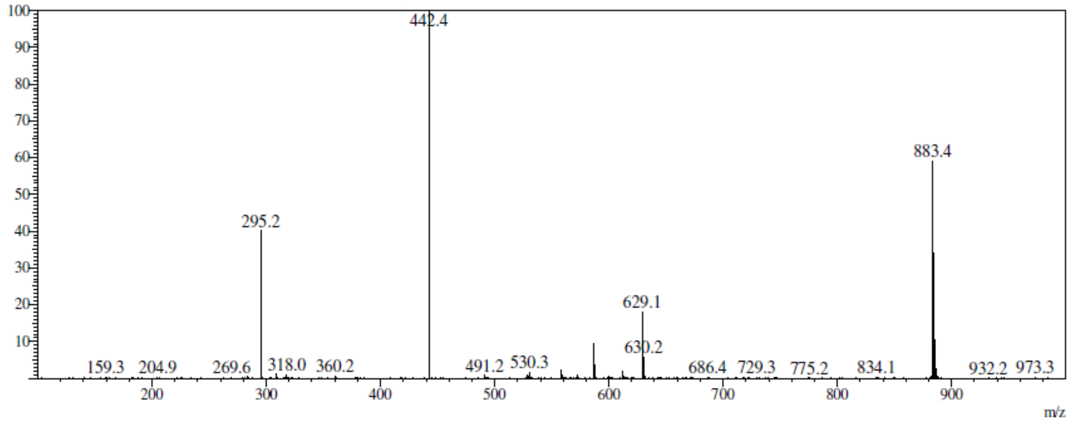

G2642 ^{1}H NMR:

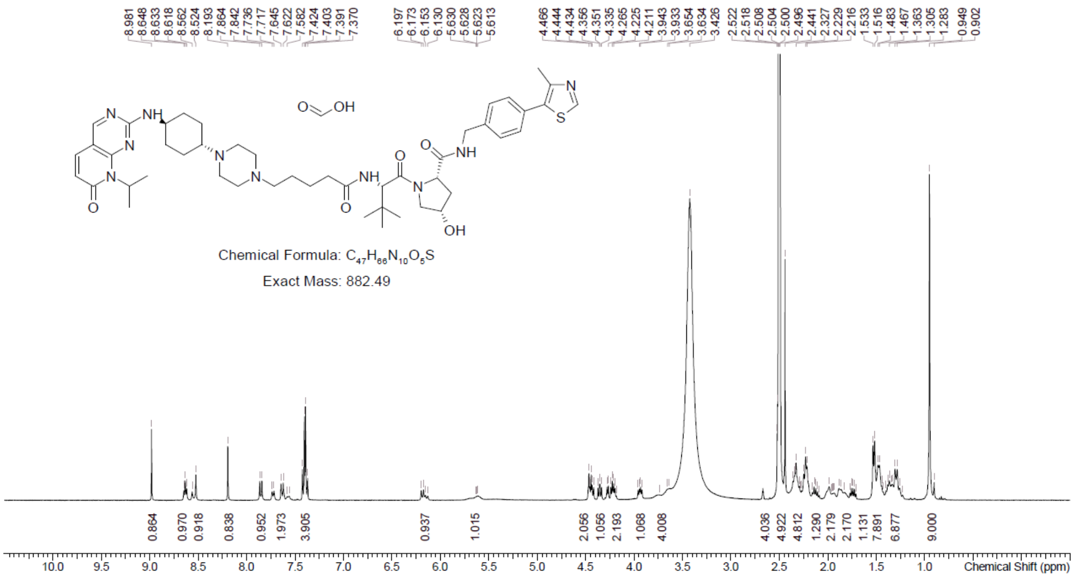

SUPPLEMENTARY REFERENCES

73. Harrington, P. E. et al. Unfolded protein response in cancer: IRE1α inhibition by selective kinase ligands does not impair tumor cell viability. *ACS Med Chem Lett* **6**, 68–72 (2015).
