# Supplementary material for: Chemically-induced degradation of the endoplasmic-reticulum stress sensor IRE1 by a VHL-recruiting chimera
Source: Nat Commun. 2025 Dec 11;16:11445. doi: 10.1038/s41467-025-66382-8 (PMC12749748; doi:10.1038/s41467-025-66382-8)
Supplement: Supplementary file 2 — Reporting Summary [file 41467_2025_66382_MOESM2_ESM.pdf]

nature portfolio

Corresponding author(s): Joachim Rudolph, PhD
Peter Hsu, PhD
Avi Ashkenazi, PhD

Last updated by author(s): Oct 27, 2025

Reporting Summary

Nature Portfolio wishes to improve the reproducibility of the work that we publish. This form provides structure for consistency and transparency in reporting. For further information on Nature Portfolio policies, see our Editorial Policies and the Editorial Policy Checklist.

Statistics

For all statistical analyses, confirm that the following items are present in the figure legend, table legend, main text, or Methods section.

n/a	Confirmed	
☐	☒	The exact sample size (n) for each experimental group/condition, given as a discrete number and unit of measurement
☐	☒	A statement on whether measurements were taken from distinct samples or whether the same sample was measured repeatedly
☐	☒	The statistical test(s) used AND whether they are one- or two-sided<br>*Only common tests should be described solely by name; describe more complex techniques in the Methods section.*
☐	☒	A description of all covariates tested
☐	☒	A description of any assumptions or corrections, such as tests of normality and adjustment for multiple comparisons
☐	☒	A full description of the statistical parameters including central tendency (e.g. means) or other basic estimates (e.g. regression coefficient) AND variation (e.g. standard deviation) or associated estimates of uncertainty (e.g. confidence intervals)
☐	☒	For null hypothesis testing, the test statistic (e.g. F, t, r) with confidence intervals, effect sizes, degrees of freedom and P value noted<br>*Give P values as exact values whenever suitable.*
☒	☐	For Bayesian analysis, information on the choice of priors and Markov chain Monte Carlo settings
☒	☐	For hierarchical and complex designs, identification of the appropriate level for tests and full reporting of outcomes
☒	☐	Estimates of effect sizes (e.g. Cohen's d, Pearson's r), indicating how they were calculated

Our web collection on statistics for biologists contains articles on many of the points above.

Software and code

Policy information about availability of computer code

Data collection	Incucyte (version 2023A Rev2)
Data analysis	CryoSPARC (version 4.6), ChimeraX (version 1.7.1), COOT (version 0.9.6) , Phenix (version 1.21), PyMOL (version 2.5.2), Prism (version 10.0.3), Grade(version 1.2.20), MSstatsTMT_2.0.1 R package, ImageJ 1.54p

For manuscripts utilizing custom algorithms or software that are central to the research but not yet described in published literature, software must be made available to editors and reviewers. We strongly encourage code deposition in a community repository (e.g. GitHub). See the Nature Portfolio guidelines for submitting code & software for further information.

Data

Policy information about availability of data

All manuscripts must include a data availability statement. This statement should provide the following information, where applicable:

- Accession codes, unique identifiers, or web links for publicly available datasets
- A description of any restrictions on data availability
- For clinical datasets or third party data, please ensure that the statement adheres to our policy

Coordinates for the IRE1:G6374:VHL complex has been deposited in the RCSB Protein Data Bank (PDB) under accession code 9N88. Corresponding electron microscopy density map has been deposited in the Electron Microscopy Data Bank (EMDB) under accession code EMD-49119. Proteomics data have been deposited to ProteomeXChange Consortium via the MassIVE partner repository with the dataset identifier MSV000097222 (https://massive.ucsd.edu/ProteoSAFe/private-dataset.jsp?task=1aa403d6dc3647fb9157d230f9d7d2f6). The authors declare that all other data supporting the findings of this study are available within the article

and its Supplementary Data files, or from the corresponding authors on request. Source data are provided with this paper.

Research involving human participants, their data, or biological material

Policy information about studies with human participants or human data. See also policy information about sex, gender (identity/presentation), and sexual orientation and race, ethnicity and racism.

Reporting on sex and gender	N/A
Reporting on race, ethnicity, or other socially relevant groupings	N/A
Population characteristics	N/A
Recruitment	N/A
Ethics oversight	N/A

Note that full information on the approval of the study protocol must also be provided in the manuscript.

Field-specific reporting

Please select the one below that is the best fit for your research. If you are not sure, read the appropriate sections before making your selection.

- [x] Life sciences
- [ ] Behavioural & social sciences
- [ ] Ecological, evolutionary & environmental sciences

For a reference copy of the document with all sections, see nature.com/documents/nr-reporting-summary-flat.pdf

Life sciences study design

All studies must disclose on these points even when the disclosure is negative.

Sample size	As stated in the figure legends
Data exclusions	As indicated in the Source data file
Replication	As stated in the figure legends
Randomization	N/A
Blinding	N/A

Reporting for specific materials, systems and methods

We require information from authors about some types of materials, experimental systems and methods used in many studies. Here, indicate whether each material, system or method listed is relevant to your study. If you are not sure if a list item applies to your research, read the appropriate section before selecting a response.

Materials & experimental systems

n/a	Involved in the study	
[ ]	[x]	Antibodies
[ ]	[x]	Eukaryotic cell lines
[x]	[ ]	Palaeontology and archaeology
[x]	[ ]	Animals and other organisms
[x]	[ ]	Clinical data
[x]	[ ]	Dual use research of concern
[x]	[ ]	Plants

Methods

n/a	Involved in the study	
[x]	[ ]	ChIP-seq
[x]	[ ]	Flow cytometry
[x]	[ ]	MRI-based neuroimaging

Antibodies

Antibodies used	Primary antibodies used in the studies: IRE1α (14C10) Rabbit mAb (Cell Signaling, #3294), GAPDH (14C10) Rabbit mAb (Cell Signaling, #2118), CDK7 Antibody (Cell Signaling, #2090), CDK9 (C12F7) Rabbit mAb (Cell Signaling, #2316), GSK-3α (D80E6) Rabbit mAb (Cell Signaling, #4337), CSF-1R/M-CSF-R Antibody (Cell Signaling, #3152), SAPK/JNK Antibody (Cell Signaling, #9252), JNK2 (56G8) Rabbit mAb (Cell Signaling, #9258), JNK3 (55A8) Rabbit mAb (Cell Signaling, #2305), MELK Antibody (Cell Signaling, #2274), PKD/PKCµ (D4J1N) Rabbit mAb (Cell Signaling, #90039), TAO2 antibody (Novus Biologicals, NBP2-20563), K48-linkage Specific Polyubiquitin Antibody (Cell Signaling, # 4289), VHL Antibody (Cell Signaling, #68547), LC3A/B (D3U4C) XP® Rabbit mAb (Cell Signaling, #12741), p-

	IRE1 antibody (made in-house at Genentech, described in Chang et al.65), K11 linkage-specific antibody (made in-house at Genentech, described in Matsumoto et al.66)
Validation	The p-IRE1 was validated in the following publication:<br><br>T. K. Chang, et al., Coordination between two branches of the unfolded protein response determines apoptotic cell fate. Mol. Cell 71, 629–636.e5 (2018).<br><br>The K11 poly-ub antibody was characterized by:<br><br>Matsumoto, M. L. et al. K11-Linked Polyubiquitination in Cell Cycle Control Revealed by a K11 Linkage-Specific Antibody. Mol. Cell 39, 477–484 (2010).<br><br>Commercial antibodies were characterized by the vendors. The inforamtion is available by searching the Cat.# on https://www.cellsignal.com/

Eukaryotic cell lines

Policy information about cell lines and Sex and Gender in Research

Cell line source(s)	AMO1, KMS27, OPM2, U2OS, Colo201, H929, HCT116, HEK293T
Authentication	All cell lines were authenticated by DNA sequencing
Mycoplasma contamination	Tested negative for mycoplasma contamination
Commonly misidentified lines (See ICLAC register)	N/A

Plants

Seed stocks	N/A
Novel plant genotypes	N/A
Authentication	N/A
